# A Comprehensive and Comparative Study on the Biochemical Composition and Non-Volatile Taste Compounds of Thirteen Shellfish Species

**DOI:** 10.3390/foods14091595

**Published:** 2025-04-30

**Authors:** Long Li, Zhiyu Fu, Yujun Liu, Zhiyuan Song, Xinrui Yang, Di Yu, Qingzhi Wang, Hai Chi, Jie Zheng

**Affiliations:** 1 Liaoning Ocean and Fisheries Science Research Institute, Dalian Key Laboratory of Genetic Resources for Marine Shellfish, Key Laboratory of Protection and Utilization of Aquatic Germplasm Resource, Ministry of Agriculture and Rural Affairs, Dalian 116023, China; liilong1995@126.com (L.L.); hkyfzy@126.com (Z.F.); lyj7005@126.com (Y.L.); wqzlm@126.com (Q.W.); chih@ecsf.ac.cn (H.C.); 2 College of Food Science and Engineering, Dalian Ocean University, Dalian 116023, China; songzhiyuan@dlou.edu.cn (Z.S.); 13030770050@163.com (X.Y.); 3 Dalian Jinshiwan Laboratory, Dalian 116034, China

**Keywords:** shellfish meat, boiling liquids, biochemical composition characteristics, non-volatile flavor profile, equivalent umami concentration

## Abstract

This study aimed to systematically investigate and compare the biochemical composition of 13 locally abundant shellfish species (Dalian, China) and the taste characteristics of these shellfish and their boiling liquids. The results showed that *Chlamys farreri* exhibited the highest level of protein (64.58%) and polyunsaturated fatty acids (53.84% of total fatty acids), whereas *Scapharca subcrenata* showed a better composition and proportion of essential amino acids (EAA/TAA = 39.02%, EAA/NEAA = 63.98%) compared to other species. Glu, Gly, Ala, Arg, 5′-monophosphate (GMP), lactic acid, succinic acid, and malic acid were quantitatively determined as the main taste compounds in shellfish and their boiling liquids. The equivalent umami concentration (EUC) values, reflecting the synergistic effect of umami compounds, showed distinct characteristics, and the maximum umami intensities were found in *Meretrix meretrix* (586.8 g monosodium glutamate (MSG)/100 g) and the boiling liquid of *Clinocardium californiense* (358.3 g MSG/100 g), respectively. Based on these experimental results, *C. californiense* was found to have the highest prehensive quality score as revealed by principal component analysis (PCA). These results are important for promoting studies aimed at nutritional value development and taste compounds improvement of these shellfish species, especially for flavor enhancer development. Meanwhile, different shellfish species can be comprehensively developed and utilized based on their distinct nutritional properties, and this would translate into greater profitability for producers.

## 1. Introduction

Shellfish are greatly valued for their rich nutritional content, umami characteristics and high cost-effectiveness. Differing from fish and crustaceans, shellfish are unfed species, relying entirely on phytoplankton and suspended organic matter found within their aquatic habitat [1,2]. According to the China Fishery Statistical Yearbook [3], shellfish aquaculture production in 2023 was 16.65 million tons, accounting for about 28.67% of overall aquaculture production in China. Oysters, clams, and scallops are the most abundant cultivated shellfish species. The total mariculture area of shellfish is 1.36 million hectares, primarily in Shandong, Fujian, and Liaoning provinces. Shellfish contain essential nutrients critical for maintaining a balanced diet. Nutritionally, most shellfish can provide high-quality proteins (>50% of dry weight, >90% digestibility) containing all essential amino acids (EAAs) required for body growth and repair [4]. They are low in calories and fat (0.96–4.5 g per 100 g serving) as compared with land-based meats (3.6–18.7 g), and they are rich in vitamins not commonly found in other meats, such as vitamin B12 (98.9 μg in steamed clams), which is important in DNA stability [5,6]. In addition, extensive research has shown that shellfish possess various biological activities, such as antioxidant, antiviral, hypoglycemic, anti-aging, promoting skin wound healing, and adjusting the organism immunity [7,8]. In Asia, particularly in China, the consumption of shellfish has increased significantly, from around 5 kg per person in the 1960 to around 36 kg per person in 2015 [9].

With the establishment of shellfish research systems and the rapid development of the prepared aquatic products industry, the market demand for shellfish is increasing continuously. Concurrently, consumers are becoming much more concerned about nutritional needs. A previous study reported the quality of three species of Black Sea bivalves with twofold higher protein content in black mussels (17.4–19.9 g/100 g) than in white clams (8.13–10.61 g/100 g) [10]. Russian researchers analyzed the proximate composition and antioxidant properties of three shellfish species on the Pacific coast of Russia, and the results showed that the three shellfish species were all rich in amino acids such as taurine and ornithine, and the *Corbicula japonica* tissue showed the highest radical scavenging activity (36.0 µg ascorbic acid/g water-soluble protein), while the *Anadara broughtonii* tissue was the lowest (13.5 µg ascorbic acid/g water-soluble protein) [11]. There are also significant differences in the biochemical composition of the same shellfish species in different sea areas. For example, the protein content of *Mytilus galloprovincialis* from the Bulgarian Black Sea (17.4 g/100 g) and Mediterranean Sea (6.58 g/100 g) was quite different [11,12,13]. In addition, shellfish contain various non-volatile taste active compounds, mainly including free amino acids (FAAs), 5′-nucleotides, and organic acids, and the taste of shellfish may be due to the interaction of various non-volatile compounds. Factors found to influence the taste of shellfish have been investigated in several studies. The results of Puértolas et al. [14] found that high pressure processing could delay sensory deterioration of oysters stored at 10 °C. Based on the results of Hirabayasi et al. [15], the equivalent umami concentrations (EUCs) of mussels from July to October were 2–3 times higher than those from other periods, indicating that the best taste of mussels in Japan was from summer to early autumn. In recent years, there has been an increased focus on the utilization of shellfish in the production of seasonings, including fermented seafood sauce. This development can be attributed to the unique taste attributes of shellfish, which have been proven to enhance the flavor profile of diverse culinary dishes [16]. All this indicated that the taste of shellfish is playing an increasingly important role in human life, but the utilization of processing by-products such as shellfish boiling liquid has been largely overlooked.

Waste released from seafood processing industries was an important factor in the organic pollution of some coastal areas [17]. Of these, boiling liquids usually contain a lot of organic pollutants and are directly discharged into the surroundings. However, there are also some valuable bio-molecules in boiling liquids [18,19]. It is widely considered that the risk of food-borne infections can be significantly reduced after cooking, but it should be noted that certain nutrients are susceptible to being degraded during the cooking process, and this could finally lead to the loss of flavorful substances and essential nutrients into the boiling liquids [5]. Furthermore, some new characteristic flavors can be formed during the heat treatment process [20]. Several attempts have been made to exploit these wastewaters. Relevant volatile compounds were screened and evaluated in the concentration of snow crab cooking effluent by simultaneous steam distillation solvent extraction/gas chromatography/mass spectrometry [21]. Tremblay et al. [22] designed and optimized a membrane process to concentrate the cooking waste waters of snow crab, and the obtained volatile compounds can undergo further processing and transformation to create a wide range of natural flavoring agents for the food industry. However, there have been few attempts to investigate the differences between boiling liquids of various shellfish species.

Therefore, the present study aimed to characterize and compare the nutritional and taste profiles (including meat yield, proximate compositions, amino acids, fatty acids, taurine, and non-volatile taste active compounds) of 13 locally abundant shellfish species and their corresponding boiling liquids in the Dalian region. The results could provide a theoretical basis for the development of shellfish processed products and their comprehensive utilization and could also provide new insight for the systematic integration of multidimensional quality data such as biochemical composition and taste.

## 2. Materials and Methods

### 2.1. Materials and Reagents

Thirteen locally abundant shellfish species, including *Mactra chinensis*, *Cyclina sinensis*, *Crassostrea gigas*, *Scapharca broughtonii*, *Ruditapes philippinarum*, *Mytilus galloprovincialis*, *Clinocardium californiense*, *Solen strictus*, *Chlamys farreri*, *Scapharca subcrenata*, *Mactra veneriformis*, *Meretrix meretrix*, and *Sinonovacula constricta* (all alive) were purchased from the New Changxing Seafood Market in Dalian, China. Samples were selected based on biological replicates (at least 30 per species) collected from three independent harvest batches in the second quarter. The shellfish samples were immediately transported to the laboratory in an insulated container with ice within an hour.

Amino acid mixed standard solution (H-type) and fatty acid methyl ester standard was purchased from Wako Pure Chemical Industries, Ltd. (Osaka, Japan) and Sigma-Aldrich (St. Louis, MO, USA), respectively. The standards of 5′-monophosphate (GMP), inosine 5′-monophosphate (IMP), adenosine 5′-monophosphate (AMP), 5′-monohydrocytidine disodium (CMP), and 5′-monophosphoric acid uridine disodium (UMP) were obtained from Shanghai yuanye Bio-Technology Co., Ltd. (Shanghai, China). Germany Dr. Ehrenstorfer GmbH (Augsburg, Germany) provided the standards of organic acids standards. The other reagents used in the present study were all of analytical grade.

### 2.2. Samples Pretreatment

Each fresh shellfish species were divided into two groups: one was freeze-dried using a freezing dryer (LG-1.0, Shenyang Aerospace Xinyang Quick Frozen Equipment Manufacturing Co., Ltd., Shenyang, China) after the shells were removed for the determination of proximate composition and flavor, and the other one was first boiled with deionized water at a ratio of 2:1 (*w*/*w*) for 10 min, and then the boiling liquids were collected and freeze-dried for flavor analysis.

### 2.3. Meat Yield and Proximate Composition

The proximate compositions including crude protein, crude fat, crude polysaccharide, and ash content of each sample were determined following the National Criterion of China GB 5009.5-2016 [23], GB 5009.6-2016 [24], GB/T 9695.31-2008 [25], and GB 5009.4-2016 [26], respectively. The meat yields were calculated as follows:

(1) $$Meatyield=\frac{m_{0}-m}{m_{0}}$$

where “*m*” is the shell weight of shellfish, g; “*m*_0_” is the total weight of shellfish, g.

### 2.4. Amino Acids (AAs) and Free Amino Acids (FAAs) Analysis

The amino acid contents of shellfish were determined according to the National Criterion of China GB 5009.124-2016 [27]. Approximately 30 mg of the sample was mixed with 15 mL of 6 mol/L HCl and transferred to a hydrolysis tube filled with nitrogen. The mixture was hydrolyzed for 22 h at 110 °C in a drying oven before being fixed to 50 mL with double distilled water (ddH_2_O). The hydrolysate was vacuum-concentrated and then dissolved with 2 mL of 2 mol/L HCl. The mixture was filtered through a 0.45 μm membrane and tested for amino acid composition and contents using an amino acid automatic analyzer (L-8900, Hitachi Co., Tokyo, Japan). The chromatographic conditions were as follows: column, ion exchange column (packed with ion exchange resin) (4.6 mm × 60.0 mm); sample volume, 20 µL; gradient elution; separation column temperature, 57 °C; reaction column temperature, 135 °C; detection wavelength, 570 nm (440 nm for proline). Tryptophan content was determined according to the National Criterion of China GB/T 15400-2018 [28]. The amino acid score (AAS) was calculated as follows:

(2) $$AAS=\frac{m_{1}}{m_{2}}$$

where “*m*_1_” is the number of milligrams of an essential amino acid per gram of protein in the sample, mg/g; *m*_2_ is the number of milligrams of that essential amino acid per gram of protein in the FAO/WHO standard model, mg/g. The essential amino acid with the lowest amino acid score is considered to be the first limiting amino acid, followed by the second limiting amino acid.

The free amino acid contents of shellfish were determined according to the method of Bi et al. [29,30]. Approximately 20 mg of the sample was dissolved with 0.02 mol/L HCl, fixed to 25 mL with ddH_2_O, and centrifuged at 3500× *g* for 10 min after ultrasonic-assisted extraction for 20 min. The supernatant was filtered through a 0.45 μm membrane and further analyzed for free amino acids using an L-8900 automatic analyzer (Hitachi Co., Tokyo, Japan). Taurine (Tau) content was determined according to the National Criterion of China GB 5009.169-2016 [31]. The shellfish sample (0.30 g) was dissolved with 40 mL ddH_2_O, and an ultrasonic-assisted extraction process (40 kHz, 200 W) was conducted at 40 °C. Potassium ferricyanide solution (500 μL, 0.15 g/mL) and zinc acetate solution (500 μL, 0.30 g/mL) were subsequently added. After adjusting to 50 mL by ddH_2_O, the mixture was centrifuged at 3500× *g* for 10 min at 4 °C. Afterward, 1 mL Na_2_CO_3_ buffer (80 mmol/L) and 1 mL dansyl chloride solution (1.50 mg/mL) were added into the supernatant (approximately 1 mL) for derivatization (2 h) away from light. Methylamine hydrochloride solution (0.1 mL) was used for the termination reaction. The supernatant was filtered through a 0.45 µm membrane and analyzed by HPLC (Agilent 1260, Agilent Technologies Inc., Santa Clara, CA, USA). The HPLC parameters were as follows: column, C18 (4.6 mm × 250 mm × 5 µm, Shiseido, Tokyo, Japan); sample volume, 10 µL; flow rate, 1.0 mL/min; detector, DAD; column temperature, 30 °C; wavelength, 254 nm; mobile phase A and B were sodium acetate buffer and acetonitrile (A:B = 7:3).

### 2.5. Fatty Acids Analysis

The fatty acid contents were determined using the method described by Saito et al. [32]. The transesterification process was performed at 70 °C for an hour after approximately 50 mg of shellfish sample was dissolved in 5 mL of 2.0% methanol–sulphuric acid in a fat extraction vial. In total, 0.75 mL of ddH_2_O and 2 mL of n-hexane were subsequently added and adequately mixed. After stratification, the supernatant was collected, and the fatty acid contents were determined by a SCION-456-Gas Chromatography (GC) (Beijing Timingtron Corporation, Beijing, China). The GC parameters were as follows: column, DB-23 (30 m × 320 µm × 0.25 µm, Agilent); sample volume, 1 µL; split injection, 10:1; injector temperature, 270 °C; carrier gas, high purity nitrogen; detector temperature, 270 °C. The oven temperature was set at 130 °C for 1 min and raised to 170 °C at 10 °C/min, then 210 °C at 2.5 °C/min and held for 2 min.

### 2.6. Nucleotides Content Assay

Nucleotides content was analyzed according to the method described by Coulier et al. [33] with slight modifications. Ultrasonic treatment (40 kHz, 200 W) was carried out for 30 min after the shellfish sample (2 g) was dissolved in 10 mL of 10% perchloric acid. The mixture was then centrifuged at 6000× *g* for 20 min at 4 °C, and the extraction procedure was repeated by adding 5% perchloric acid into the sediment. All the supernatants were mixed, neutralized (pH 6.5) with 1 mol/L KOH, fixed to 50 mL with ddH_2_O, filtered through a 0.45 μm filter membrane, and then analyzed by HPLC (Agilent 1260, Agilent Technologies Inc., Santa Clara, CA, USA). The HPLC parameters were as follows: column, C18 (4.6 mm × 250 mm × 5 µm, Agilent); sample volume, 10 µL; flow rate, 1.0 mL/min; detector, DAD; column temperature, 25 °C; wavelength, 254 nm; mobile phase A and B were phosphate buffer and methanol (A:B = 1000:40).

### 2.7. Organic Acids Content Assay

Organic acids content was measured according to the previous method described by Liu et al. [34]. The sample (1 g) was ultrasonically dissolved (40 kHz, 200 W) in 5 mL of 10 mmol/L K_2_HPO_4_ (pH 2.5) for 30 min before immersion in a water bath at 60 °C for 1 h. The supernatants were collected after centrifugation at 6000× *g* for 20 min at 4 °C, filtered through a 0.45 μm membrane, and analyzed by HPLC (Agilent 1260, Agilent Technologies Inc., Santa Clara, CA, USA). The chromatographic conditions were as follows: column, SB-Aq (4.6 mm × 250 mm × 5 µm, Agilent); sample volume, 10 µL; flow rate, 0.5 mL/min; detector, DAD; column temperature, 30 °C; wavelength, 210 nm; mobile phase, 10 mmol/L K_2_HPO_4_ (pH 2.55).

### 2.8. Taste Activity Values (TAVs)

TAVs were determined by the ratio of certain taste compounds (FAAs, nucleotides, and organic acids) in the sample to their respective threshold values [35]. TAVs could indicate the characteristic contribution of a single compound to the overall taste. Compounds with TAVs > 1 were usually considered to have a significant effect on the food taste, and the higher the TAVs, the greater the contribution to food taste.

### 2.9. Equivalent Umami Concentrations (EUCs)

EUCs (g monosodium glutamate (MSG)/100 g) were defined as a synergistic combination of 5′-nucleotides (IMP, GMP, and UMP) and umami amino acids (Asp and Glu) that produced an umami intensity equivalent to that of a single MSG, as indicated by the following formula [36]:

(3) $$EUC=\sum a_{i}b_{i}+1218\left(\sum a_{i}b_{i}\right)\left(\sum a_{j}b_{j}\right)$$

where *a_i_* is the concentration (g/100 g) of each umami amino acid; *b_i_* is the relative umami concentration (RUC) for each umami MSG-like amino acid (Glu, 1 and Asp, 0.077), *a_j_* is the 5′-nucleotide concentration (g/100 g), *b_j_* is the RUC for each umami 5′-nucleotide to IMP (IMP, 1.00; GMP, 2.30; and AMP, 0.18) and 1218 is a synergistic constant.

### 2.10. Statistical Analysis

The data were presented as mean ± standard deviation (*n* ≥ 3), and one-way ANOVA and significance were established with a *p* < 0.05 using SPSS 20.0 (IBM, Chicago, IL, USA). Origin 2021 (OriginLab, Northampton, MA, USA) was used for plotting and correlation analysis. Principal component analysis (PCA) was performed by SPSS 20.0.

## 3. Results and Discussion

### 3.1. Meat Yield and Proximate Compositions

There were significant interspecies variations in meat yield and nutritional profiles among 13 shellfish species (Table 1). *M. chinensis* had a maximum meat yield (52.44%), exceeding low-yield species (*C. gigas*: 11.57%, *M. galloprovincialis*: 13.41%, *M. meretrix*: 11.93%) by 74–77%, potentially linked to the filter-feeding capacity [2,37,38]. Some pollutants in the marine environment are more likely to accumulate in shellfish, potentially resulting in impaired reproduction and limited food intake, which may further impact the meat yield [37]. In addition, growth phenotype of shellfish is influenced not only by genes but also by metabolic pathways [39], these variations can result in differing meat yields among various shellfish species. Studies by Desai et al. [40] also showed that the diverse flux of food particles and local water dynamics in these habitats may be key factors influencing the partitioning of shellfish, which may indirectly affect the meat yield of shellfish. Proximate composition analyses indicated that protein constituted the predominant component in all samples (41.24–64.58% of dry weight) and identified four protein-dominant species: *M. chinensis* (64.50%), *C. californiense* (64.32%), *S. strictus* (64.33%), and *C. farreri* (64.58%), which were significantly higher (*p* < 0.05) than that of other species and about 36% higher than that of *C. sinensis*. According to the results of Song et al. [4], the protein content of *Anadara kagoshimensis* collected from the Kerch Strait in Eastern Europe was as high as 81.8%, while only 28.9% was observed in *Crassostrea hongkongensis* from China. Crude fat contents were all lower than 10%, being consistent with the nutritional advantages of shellfish. Shellfish with high fat content are relatively uncommon, with only a limited number of studies reported, including wild *Laternula elliptica* (11.9 ± 2.7%, of wet weight) [41] and wild *Arca noae* (8.58 ± 0.02%, of wet weight) [42]. The crude polysaccharide levels exhibited the greatest interspecies variation (*p* < 0.05), with *S. constricta* (25.41%) demonstrating 4.8-fold higher glycosaminoglycan concentrations than *M. chinensis* (4.37%). The content of ash ranged from 11.25% (*S. broughtonii*) to 37.62% (*C. sinensis*).

Many factors, such as species, diet, culture depth, and culture areas, could influence the nutrition profiles of shellfish [11,43,44]. In addition, salinity can influence its nutritional composition and meat yield by modulating respiratory metabolism, glycolysis, lipolysis, and apoptosis [45]. Greater water depths of shellfish also affects growth and nutritional indicators by reducing the available food for shellfish [43]. In general, *M. chinensis* exhibited characteristics of high protein and the lowest fat content among the studied samples, making it a potentially suitable choice for a healthy human diet from a macro-nutrients perspective. Furthermore, *C. sinensis* and *M. veneriformis* showed characteristics with high ash levels, which indicated that they were rich in inorganic substances such as minerals and could also be good sources of mineral supplementation for the body. The polysaccharide content was rich in *S. constricta* and has a number of health benefits [46] that can be used in the development of functional foods.

### 3.2. Amino Acids Analysis

**Figure 1:**
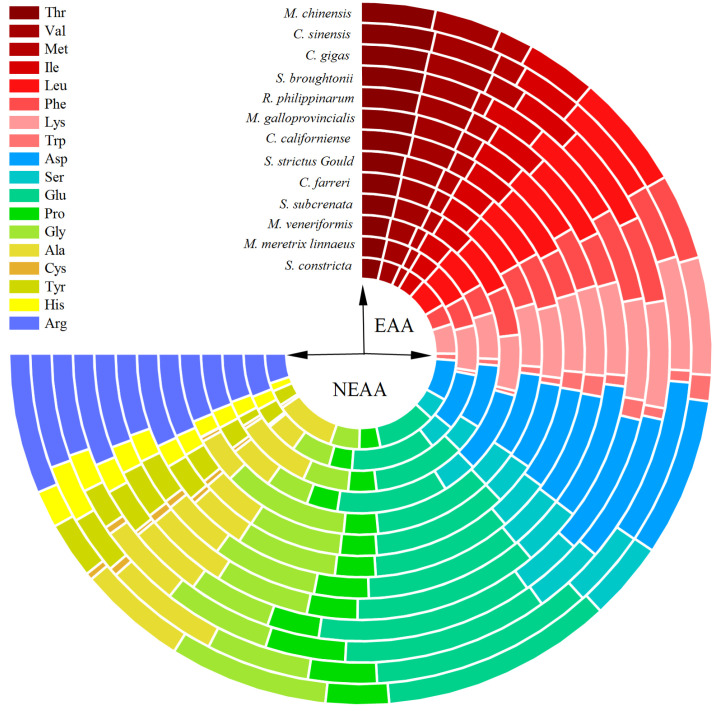
The amino acids composition of 13 shellfish species (g/100 g on a dry basis).

Amino acids (AAs) constitute the fundamental structural units of proteins, and the quality of proteins predominantly depends on their amino acid composition, in which AAs are crucial for metabolic regulation, signal transduction, and immune modulation [47]. As shown in Table 2 and Figure 1, Glu and Asp were the most abundant amino acids in all shellfish samples, followed by Arg, Lys, Ala, Leu, and Gly, which were generally observed in most shellfish species [48,49]. However, Asha et al. [50] found that Lys (14.3%) was the highest present in oyster *Crassostrea madrasensis*, followed by Thr (12.3%) and Asp (11.8%). The contents of total amino acids (TAAs) in all samples ranged from 30.03 g/100 g (*C. sinensis*) ~54.11 g/100 g (*S. strictus*) with significant differences (*p* < 0.05) between species. [51]. Song et al. [4] suggested that the amino acids profile of shellfish appear to be influenced by temporal or spatial elements rather than by the specific type of shellfish. A good example is that *Crassostrea madrasensis* collected from Moothakunnam, Kerela was rich in AAs (99.3 g/100 g Protein) [50], whereas *Crassostrea madrasensis* collected from Sattar Island, India was relatively poor (20.3 g/100 g Protein) [52]. Based on the results of this study, different shellfish species from same water have similar AAs profiles. Similar results were also found by Karaulova et al. [11], which concluded that the top five AAs of *Corbicula japonica*, *Spisula sachalinensis* and *Spisula sachalinensis* collected from coastal areas of Far East Russia are Leu, Lys, Ile, Thr and Val.

The highest EAA/TAA and EAA/NEAA (nonessential amino acid) values were observed in *S. subcrenata* (39.02% and 63.98%, respectively), which were the closest to the FAO/WHO recommended normative values (40% and 60%), followed by *S. broughtonii* (38.30% and 62.08%) and *C. gigas* (37.93% and 61.11%). The nutritional quality of food proteins relies heavily on the composition and proportion of their essential amino acids [53]; therefore, amino acid scoring (AAS) was used to evaluate the proteins of 13 shellfish species. As shown in Table 3, almost all AAs were lower than the reference value. A similar result was also found in many other studies, such as *Saccostrea cuccullata*, *M. meretrix*, and *Crassostrea virginica* collected from Bangladesh [51] and *Hyotissa hyotis* collected from Jeju Island, Korea [54]. The first limiting amino acid in most shellfish was Met and Cys, which ranged from 0.33~0.68, and this was in accordance with the finding reported by Song et al. [55]. Cys and Met, collectively referred to as sulfur-containing amino acids, can delay the aging process and aging-related diseases and improve glucose [56]. The second limiting amino acid of *M. chinensis*, *S. broughtonii*, *M. galloprovincialis*, *C. californiense*, *M. veneriformis*, and *S. constricta* was Val, while that of *C. sinensis*, *R. philippinarum*, *S. strictus*, and *M. meretrix* was Trp. Hence, it is necessary to provide targeted nutritional reinforcement to ensure its nutritional function during shellfish product development.

### 3.3. Fatty Acids Composition

**Figure 2:**
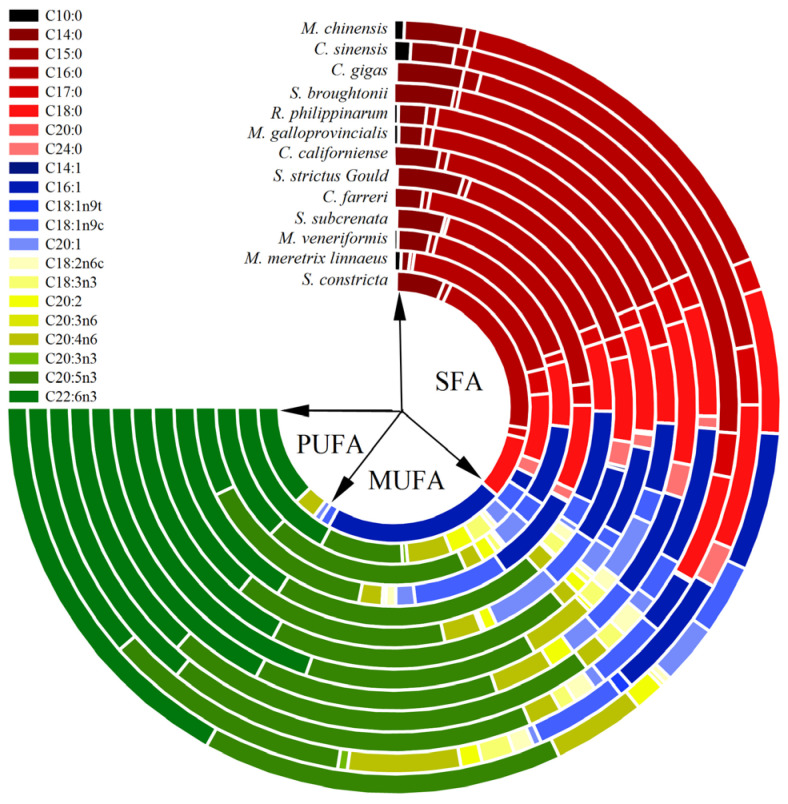
The fatty acids composition of 13 shellfish species (% of total fatty acids).

In this study, a total of 21 fatty acids were identified in 13 shellfish species (Table 4 and Figure 2). The identified number of saturated fatty acids (SFAs) was eight, accounting for 33.71% in *C. farreri* to 48.35% in *S. constricta*, which is in agreement with the studies reported by Valenzuela et al. [57]. The major SFA was C16:0 and constituted more than half of the SFAs, and another highly abundant fatty acid was C18:0 (6.01~10.23%). Similar results have also been reported in *Anodonta pseudodopsis* and *Unio tigridis* from the South eastern Mediterranean region of Turkey [58]. In the same way, the monounsaturated fatty acids (MUFAs) number was five and was the least abundant of the fatty acids. Similarly, Zhu et al. [59] also observed MUFAs representing 14.53% to 16.64% in different shell color strains of *C. gigas*, significantly lower than SFAs and polyunsaturated fatty acids (PUFAs). PUFAs are the most extensively studied fatty acids due to their significant physiological roles and health implications [60]. It has commonly been assumed that the content of PUFAs in marine organisms was generally higher than that of SFAs and MUFAs [61]. PUFAs were eight and around 50% (except for *C. sinensis*, *S. subcrenata*, and *S. constricta*). The profiles of PUFAs in *C. farreri* (53.84%), *M. galloprovincialis* (52.85%), *C. californiense* (52.00%), *S. broughtonii* (51.93%), and *M. chinensis* (50.23%) all exceed 50%. In contrast, the profiles of PUFAs in *S. constricta* (19.95%) were significantly lower (*p* < 0.05) than that of other samples, quite different from the results of Ran et al. [62]. This may be related to factors such as diet and salinity, both of which have been shown to affect the profiles of fatty acids of *S. constricta* [62,63]. C20:5n3 (11.45~29.10%, EPA) and C22:6n3 (9.70~26.59%, DHA) were the major PUFAs, consistent with Hu’s studies in *Patinopecten yessoensis* and *Chlamys farreri* from Dalian, Liaoning, China [64], which supported the results of the present study.

Shellfish are abundant in polar lipids, primarily phospholipids and sphingolipids [65]. EPA and DHA in PUFAs are the primary fatty acids found in phospholipids, which have preventive effects on cardiovascular disease and other inflammatory conditions [66,67]. EPA and DHA are also critical indicators for evaluating the nutritional value of lipids. The profile of EPA in *S. broughtonii* (29.10%) was significantly higher than that of other shellfish (*p* < 0.05), followed by *C. californiense* (25.29%), *C. farreri* (27.26%), and *M. veneriformis* (25.25%). Consistent with this study, Liu et al. [68] found that EPA content was significant higher in *S. broughtonii* compared to *S. subcrenata* from the Yellow Sea in July in Dalian (*p* < 0.05). The profile of DHA in *M. galloprovincialis* (26.59%) was the highest, while *S. subcrenata* was 9.70%, which was much lower than other shellfish (*p* < 0.05). Furthermore, the n-3/n-6 PUFAs ratio recommended by the World Health Organization is 1:1 or above [69], which is considered as an important determinant to evaluate the nutritional value [70]. In this study, all shellfish species provided a high n-3/n-6 PUFAs ratio (between 4.15 for *C. sinensis* and 25.82 for *C. californiense*), which was considered to be favorable from a nutritional perspective. This is a common observation in many other marine organisms, as n-3 PUFAs (primarily EPA and DHA) are plentiful compared to n-6 PUFAs in the marine food chain [71]. Rincón-Cervera et al. [72] also detected a higher n-3/n-6 PUFAs ratio in clam (*Venus antiqua*, 11.1) and razor clam (*Mesodesma donacium*, 25)

### 3.4. Free Amino Acids Analysis

The FAAs are closely linked to flavor and hold significant importance in shellfish. These FAAs have varying taste characteristics, including umami, sweetness, and bitterness, due to their different structures [73]. As demonstrated in Table 5, a divergence in the content and composition of FAAs was observed between the shellfish and their respective boiling liquids. The highest content of total free amino acids was detected in *S. constricta* (14.09 g/100 g), which was 2.5 times higher than that of *C. sinensis* (5.25 g/100 g). Tremblay et al. [17] reported that FAAs content of freeze-dried lobster (*Homarus americanus*) retentate was 11.75%. Hirabayasi et al. [15] investigated the seasonal variations in extractive components in *Mytilus galloprovincialis* mussels; the results showed that FAAs content ranged from 1.15 g/100 g in November to 2.03 g/100 g in August of wet weight. The most abundant FAAs in the majority of shellfish were Gly, Arg, Ala, and Glu, which significantly influenced the flavor profile. These results are similar to those reported by Song et al. [55]. The researchers found that the highest content of FAAs in *C. farreri* was Gly, up to 293.26–369.94 mg/100 g of wet weight, followed by taurine, ranging from 216.4 to 241.91 mg/100 g. Taurine is a free non-protein amino acid that plays an important role in promoting neuronal development [74] and early treatment of cancer [75]. This study also found high levels of taurine in 13 shellfish species, ranging from 0.56 g/100 g in *C. farreri* and *S. constricta* to 1.32 g/100 g in *S. subcrenata*. The human body has a low ability to synthesize taurine on its own, so the abundant content of taurine in marine organisms such as shellfish can serve as a good source for the human body to obtain taurine. The umami FAAs (Asp and Glu) in the *M. meretrix* (2.05 g/100 g) were significantly higher than that of other shellfish (*p* < 0.05). As for the sweet FAAs, the summation content in *C. farreri* (11.14 g/100 g) was significantly higher than that in other shellfish (*p* < 0.05). As for the bitter FAAs, the highest content was detected in *S. broughtonii* (2.87 g/100 g).

As depicted in Table 6, the boiling liquids of *S. constricta* (26.95 g/100 g) and *C. farreri* (24.06 g/100 g) exhibited the highest levels of total FAAs, surpassing those found in the other shellfish species. The boiling liquid of *S. broughtonii* (3.92 g/100 g) has the lowest level of FAAs compared to others. It was also found that the FAAs content of most boiling liquids was higher than that of fresh shellfish. The protein in the shellfish meat is degraded into smaller molecules of peptides and amino acids during the boiling process, which subsequently dissolves into the boiling liquids [76]. And the content of total free amino acids gradually increases with the rise in temperature [77]. Furthermore, different shellfish exhibit significant variations in protein decomposition rate and water diffusion rate under identical heating conditions due to their distinct structural characteristics [78]. The levels of umami, sweet, and bitter FAAs in boiling liquids of various shellfish ranged from 1.26 g/100 g to 3.34 g/100 g, 1.36 g/100 g to 22.68 g/100 g, and 0.47 g/100 g to 2.69 g/100 g, respectively. Notably, the level of sweet FAAs was significantly higher than that of umami and bitter FAAs in both shellfish and boiling liquids, which function palatably in taste. These results are consistent with those of other reports. For instance, the sweet FAAs identified in oysters by Liu et al. [77], namely Ala, Gly, Glu, and His, accounted for 76.79~80.24% of the total FAAs.

**Figure 3:**
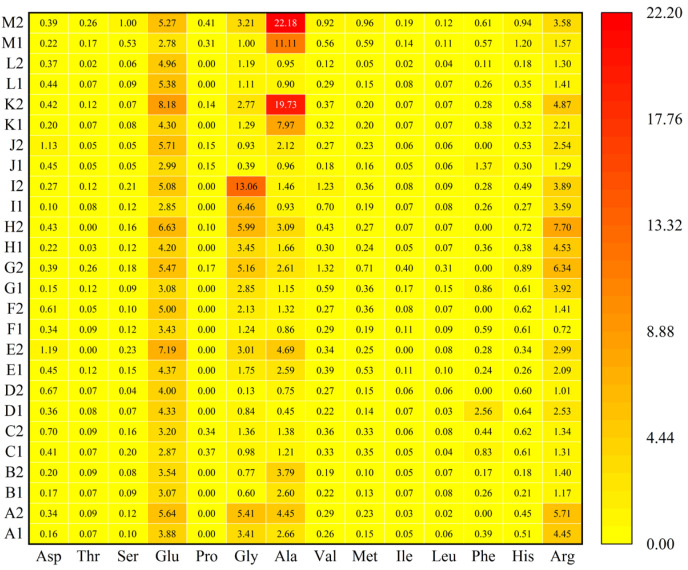
Heatmap of TAVs of free amino acids. A to M are *M. chinensis*, *C. sinensis*, *C. gigas*, *S. broughtonii*, *R. philippinarum*, *M. galloprovincialis*, *C. californiense*, *S. strictus*, *C. farreri*, *S. subcrenata*, *M. veneriformis*, *M. meretrix* and *S. constricta*, respectively. The numbers 1 and 2 represent shellfish meat and boiling liquids, respectively. The taste threshold (mg/100 mL) was taken from the literature [80].

The TAVs of each FAA in shellfish meat and their boiling liquids were calculated to assess the impact of FAAs on taste. The results presented in Figure 3 indicated that almost all TAVs of FAAs were below 1, except for Glu, Gly, Ala, and Arg. It suggested that Glu, Gly, Ala, and Arg had a significant impact on enhancing the flavor profile of shellfish meat and their boiling liquids. The results in this research were similar to those reported by Bi et al. [79], who found that the TAVs of Glu, Gly, and Ala in Pacific oysters were higher than 1. Obviously, the TAVs corresponding to each FAAs in boiling liquids were higher than those of shellfish meat, which exhibited potential applications for developing liquid formulations.

### 3.5. 5′-Nucleotides Analysis

5′-nucleotides, including 5′-GMP, 5′-IMP, 5′-AMP, 5′-CMP, and 5′-UMP, are key flavor compounds found in seafood [81]. Notably, GMP, IMP, and AMP contribute significantly to the umami taste [82] and function with MSG and umami amino acids to improve the overall umami taste of food [36]. However, the umami tastes of UMP and CMP are not good on their own, requiring the assistance of monosodium glutamate for better flavor [80]. These nucleotides, thus, contribute to enhancing the umami taste.

As shown in Table 7 and Table 8, the primary nucleotide found in various shellfish species was GMP (15.56~151.51 mg/100 g), and their TAVs all exceeded 1, making it one of the crucial factors contributing to the umami taste of shellfish. *R. philippinarum* (52.57 mg/100 g) and *M. meretrix* (63.10 mg/100 g) were also abundant in IMP, with TAVs exceeding 1. Zhang et al. [83] also found that GMP and IMP were the main nucleotides in oyster hydrolysates treated by different methods. Currently, the “IMP + GMP” has been effectively utilized as a flavor enhancer in various food products, including oyster sauce, soup stocks, and so on [84]. Therefore, the high “IMP + GMP” content of *M. meretrix* gives it the potential to be developed as a flavor enhancer. In contrast to this study, Liu et al. [85] reported that AMP content (9.76–186.94 mg/100 g) was highest in scallop (*Patinopecten yessoensis*) collected from different seasons. Dong et al. [86] found extremely low levels of AMP in the body trunk, gill, and mantle of the *C. gigas*, and AMP content in adductor muscle tend to increase and then decrease during storage. In another report, AMP content is also related to the thawing method [87].

As for various boiling liquids, the predominant nucleotide was UMP (22.62~125.87 mg/100 g), followed by GMP (8.51~64.02 mg/100 g). Additionally, the TAVs of GMP in all boiling liquids (except *C. sinensis* and *S. broughtonii*) exceeded 1, significantly influencing their umami taste. In contrast, AMP and CMP were detected at low levels or were undetectable in various shellfish meat and their boiling liquids.

### 3.6. Organic Acids Analysis

Organic acids play a crucial role in the flavor profile of aquatic products as their composition and content are closely linked to the metabolic processes of organisms. Furthermore, the effects of organic acids encompass some important functions in the human body, including anti-inflammatory, osteoporosis prevention, immune function regulation, promotion of calcium absorption, and anti-obesity capabilities [88].

**Table 8:** TAVs of nucleotides and organic acids in 13 shellfish meat and their boiling liquids.

		GMP	IMP	AMP	Lactic Acid	Succinic Acid	Malic Acid
Taste Threshold/(mg/100 mL)		12.5	25	50	126	37	50
Shellfish meat	*M. chinensis*	2.68	0.32	-	1.51	36.87	83.06
	*C. sinensis*	2.55	0.33	-	0.03	34.78	36.61
	*C. gigas*	3.39	0.28	-	12.33	-	53.61
	*S. broughtonii*	1.24	-	-	11.30	53.09	47.71
	*R. philippinarum*	4.60	2.10	0.03	30.66	10.95	101.59
	*M. galloprovincialis*	12.12	-	0.03	26.40	9.35	29.89
	*C. californiense*	7.99	-	-	8.10	28.21	30.15
	*S. strictus*	5.74	-	-	3.25	24.95	271.49
	*C. farreri*	3.34	0.02	-	12.48	-	20.83
	*S. subcrenata*	2.55	-	0.05	5.17	36.61	28.55
	*M. veneriformis*	3.40	0.15	0.09	15.08	24.99	65.05
	*M. meretrix*	7.92	2.52	0.03	-	41.67	87.55
	*S. constricta*	2.06	-	-	26.41	26.67	114.01
Boiling liquids	*M. chinensis*	1.36	0.31	-	3.39	37.56	90.88
	*C. sinensis*	0.68	0.08	-	2.87	33.92	64.50
	*C. gigas*	1.30	0.13	-	7.05	9.15	49.52
	*S. broughtonii*	0.74	-	-	3.83	19.86	87.52
	*R. philippinarum*	2.02	1.83	-	3.55	3.73	50.29
	*M. galloprovincialis*	2.25	0.39	-	37.79	6.69	31.80
	*C. californiense*	5.12	1.13	-	34.60	10.21	253.63
	*S. strictus*	1.83	0.15	-	5.13	40.65	506.00
	*C. farreri*	3.80	0.86	-	4.88	29.42	115.18
	*S. subcrenata*	1.55	0.21	-	4.06	59.55	196.23
	*M. veneriformis*	2.28	0.39	-	9.44	100.05	168.01
	*M. meretrix*	1.00	0.23	-	2.26	115.65	142.52
	*S. constricta*	3.12	0.19	-	25.59	-	354.28

- not detected. Taste threshold (mg/100 mL) was taken from the literature [80,89].

A total of eight organic acids were detected in 13 shellfish meat and their boiling liquids (Table 9). The main organic acids in all samples were tartaric acid, lactic acid, malic acid, and succinic acid. An increased concentration of organic acids can reduce umami perception [88]. However, there are not sufficient perceptual data to draw definitive conclusions about some interactions. The analysis revealed that the levels of total organic acids present in the majority of the cooking liquids exceeded those found in the shellfish meat, with the content in cooking liquids of *C. californiense* exhibiting an approximately two-fold increase. The TAVs of lactic acid, succinic acid, and malic acid exceeded 1 in almost all samples. Among these, malic acid had the greatest impact on the taste, followed by succinic acid, and this was consistent with the results of Zhu et al. [89]. Previous research found that the addition of malic acid to low-sodium salt reduced the bitterness introduced by potassium salt and improved the salty flavor [90].

### 3.7. EUC Analysis

**Figure 4:**
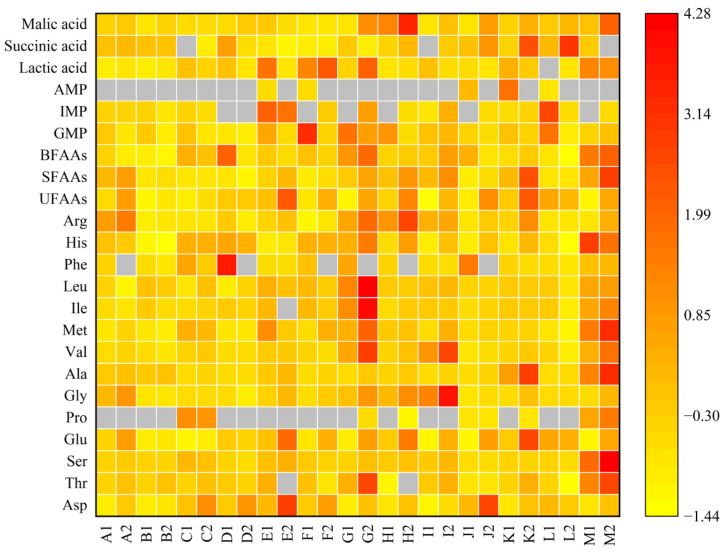
Heatmap of TAVs of different taste compounds. A to M are M. chinensis, C. sinensis, *C. gigas*, S*. broughtonii, R. philippinarum*, *M. galloprovincialis*, *C. californiense*, *S. strictus*, *C. farreri*, *S. subcrenata*, *M. veneriformis*, *M. meretrix* and *S. constricta*, respectively. The numbers 1 and 2 represent shellfish meat and boiling liquids, respectively.

A heatmap of tastants was constructed to visualize each component of taste quality (Figure 4). The closer the color block was to red, the higher the TAVs corresponding to the taste compounds, and the closer it was to yellow, the lower the TAVs. The gray color block indicated that the component was not detected. The contents of flavoring substances were higher in *S. constricta* and boiling liquids of *C. californiense* and *S. constricta*, as evidenced by the predominant red distribution in the heatmap. Although a TAV can evaluate the contribution of a single component to taste, it does not take account of the interactions between various components of food, including synergy or masking effects [91]. EUC was used to quantify the synergistic effect of umami FAAs and 5′-nucleotides [82]. As shown in Table 10, *M. meretrix* had the highest value (586.8 g MSG/100 g), followed by *M. galloprovincialis* (450.4 g MSG/100 g), while *S. broughtonii* (59.4 g MSG/100 g) and *S. constricta* (62.3 g MSG/100) were significantly lower than those of the other samples (*p* < 0.05). For boiling liquids, the EUC of *C. californiense* (358.3 g MSG/100 g) was significantly higher than that of the other samples (*p* < 0.05) and 12 times as high as *C. sinensis* (29.1 g MSG/100 g). *M. meretrix* exhibited the highest EUC among 13 shellfish species, while EUC of *M. meretrix* boiling liquid was found to be low. It suggested that the high intensity of umami taste in shellfish may not necessarily correspond to their boiling liquids. It has also been observed that the EUC results are larger than those obtained by other researchers. This discrepancy can be attributed to the different baselines (of dry weight or wet weight) for the calculation of indicators.

### 3.8. PCA

**Figure 5:**
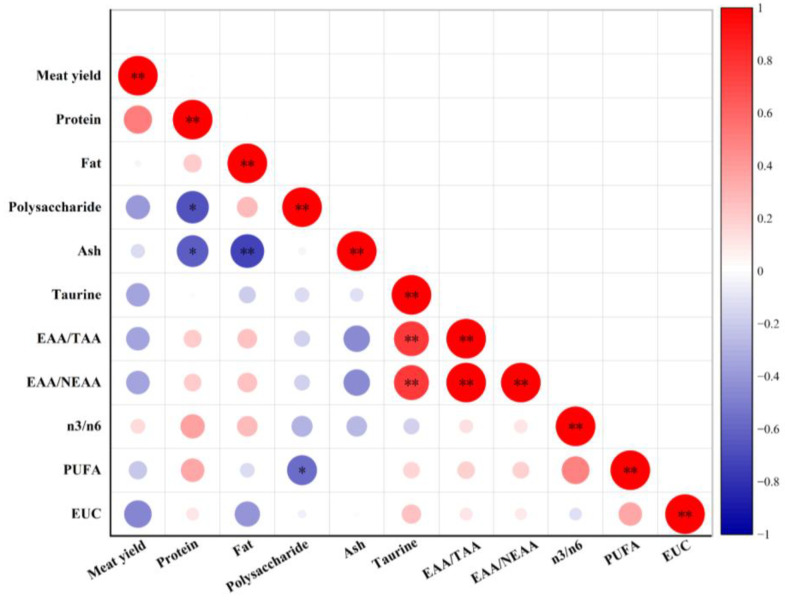
Correlation analysis among nutritional and flavor indicators. * *p* < 0.05, ** *p* < 0.01.

**Figure 6:**
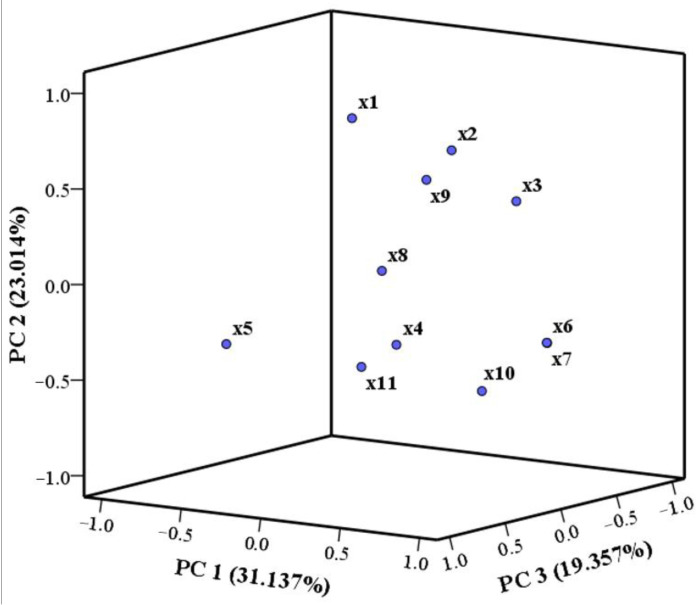
Three-dimensional PCA plot of the 11 quantitative traits with regard to the first three principal components.

PCA is a commonly used statistical method in data analysis, with the primary purpose of identifying the potential combinations of traits or variables that account for the majority of variance in a given dataset [92]. Before PCA, Pearson correlation analysis was used to reveal the association between the variables [93]. There was an absence of correlation between the majority of the indicators (Figure 5). Consequently, PCA was employed to reduce the dimension of these experimental parameters. Four principal components that contributed to the total variation were identified (Table 11). The eigenvalues of PC1 to PC4 were all larger than 1, and these four PCs explained a total variance of 84.478% in the analysis, which can efficiently reflect the original data in the indices of samples. The larger the absolute value of the loading of variables, the greater its impact on PCs. The variance contribution rate of PC1 was 31.137%, which was primarily responsible for the differences in the EAA/TAA, EAA/NEAA, taurine, and ash. The meat yield and protein primarily contribute to the formation of PC2 (variance contribution rate of 23.014%). The variance contribution rate of PC3 accounted for 19.357%, and it was found to be strongly associated with fat, PUFA, and polysaccharide. PC4 primarily indicated the levels of meat yield and n3/n6, with a variance contribution rate of 10.970%. Additionally, a PCA scatter plot was created with the first three principal components (Figure 6).

PCs were expressed as the following formulas based on the PCs’ load matrix. Subsequently, the comprehensive quality score, which serves as a quality evaluation model for shellfish, was obtained in Equation (8):

(4) $$\begin{array}{r} F1=-0.120\times V_{1}+0.289\times V_{2}+0.154\times V_{3}-0.204\times V_{4}-0.336\times V_{5}+0.491\times V_{6}\\ +0.488\times V_{7}+0.244\times V_{8}+0.180\times V_{9}+0.371\times V_{10}+0.121\times V_{11} \end{array}$$

(5) $$\begin{array}{r} F2=0.500\times V_{1}+0.460\times V_{2}+0.191\times V_{3}-0.311\times V_{4}-0.217\times V_{5}-0.178\times V_{6}\\ -0.181\times V_{7}+0.104\times V_{8}+0.344\times V_{9}-0.327\times V_{10}-0.239\times V_{11} \end{array}$$

(6) $$\begin{array}{r} F3=-0.018\times V_{1}+0.108\times V_{2}-0.534\times V_{3}-0.445\times V_{4}+0.365\times V_{5}-0.110\times V_{6}\\ -0.119\times V_{7}+0.456\times V_{8}+0.067\times V_{9}+0.072\times V_{10}+0.359\times V_{11} \end{array}$$

(7) $$\begin{array}{r} F4=-0.464\times V_{1}-0.121\times V_{2}+0.317\times V_{3}+0.267\times V_{4}-0.045\times V_{5}-0.122\times V_{6}\\ -0.128\times V_{7}+0.396\times V_{8}+0.487\times V_{9}-0.329\times V_{10}+0.252\times V_{11} \end{array}$$

(8) $$F=0.311\times F_{1}+0.230\times F_{2}+0.194\times F_{3}+0.110\times F_{4}$$

where the coefficients in Equation (4) to Equation (7) are the eigenvectors of the indices, V_1_ to V_11_ are the standardized values of indices, respectively, and F1, F2, F3, and F4 are the scores of the PCs. The coefficients in Equation (8) are the variance contribution rates of the initial eigenroots.

The comprehensive quality scores of 13 shellfish species were determined using Equation (8) (Table 12). From the PCA-based mathematical model, the highest scores of 13 shellfish species were *C. californiense* (1.18), followed by *S. broughtonii* (0.64), *C. farreri* (0.64), and *M. galloprovincialis* (0.54), which meant these shellfish had a greater comprehensive quality.

## 4. Conclusions

This study has systematically revealed the interspecies variability in nutritional and flavor profiles among 13 commercially significant shellfish species from Dalian, China, providing practicable insights for species-specific utilization. The results showed that each shellfish species had unique nutritional characteristics, and the comprehensive quality of *C. californiense* was relatively the best. Specifically, *M. chinensis* had the highest meat yield and was likely to be more economically and productively advantageous. *C. farreri* and *S. subcrenata* emerged as the optimal protein source, with the highest protein content and the most balanced amino acid profile, respectively. Despite the low fat contents (less than 10%), all shellfish species exhibited a nutritionally beneficial n-3/n-6 PUFA ratio, highlighting their potential as a functional food for cardiovascular health. *M. meretrix* exhibited the maximal EUC values and was, therefore, considered to be more acceptable than the other shellfish species. In addition, the higher EUC values of the *C. californiense* boiling liquid showed its viability as a natural umami enhancers in reduced-sodium products. Although the present study provided systematic and comprehensive information about the nutritional quality and taste characteristics of 13 common shellfish species, many factors, such as the time elapsed from fishing to market arrival, storage methods, transportation conditions, and fishing seasons, which can influence the quality characteristics of these shellfish species, were not properly involved in the present study. To further develop and utilize the shellfish resources more precisely, their nutritional and processing characteristics require a more systematic and in-depth study, possibly focusing on the identification of crucial quality factors and the elucidation of quality formation mechanism by multi-omics and other newly developed technologies.

## Figures and Tables

**Table 1:** Meat yield and proximate composition (% on a dry basis, *n* = 3) of 13 shellfish species.

	Meat Yield	Crude Protein	Crude Fat	Crude Polysaccharide	Ash
*M. chinensis*	52.44	64.50 ± 0.55 ^a^	2.05 ± 0.09 ^e^	4.37 ± 0.20 ^h^	23.93 ± 0.04 ^c^
*C. sinensis*	33.10	41.24 ± 0.82 ^f^	1.83 ± 0.18 ^e^	15.03 ± 0.23 ^d^	37.62 ± 0.33 ^a^
*C. gigas*	11.57	45.82 ± 1.00 ^e^	5.65 ± 0.37 ^b^	20.93 ± 0.54 ^b^	18.24 ± 0.53 ^e^
*S. broughtonii*	32.43	61.02 ± 0.39 ^b^	7.72 ± 0.18 ^a^	15.63 ± 0.53 ^d^	11.25 ± 018 ^h^
*R. philippinarum*	34.04	57.45 ± 0.57 ^c^	3.46 ± 0.29 ^cd^	19.38 ± 0.71 ^bc^	15.08 ± 0.52 ^f^
*M. galloprovincialis*	13.41	61.77 ± 0.95 ^b^	4.27 ± 0.77 ^c^	9.70 ± 0.38 ^fg^	19.32 ± 0.18 ^e^
*C. californiense*	41.49	64.32 ± 0.52 ^a^	4.38 ± 0.36 ^c^	8.72 ± 0.37 ^g^	17.90 ± 0.14 ^e^
*S. strictus*	48.62	64.33 ± 0.28 ^a^	3.32 ± 0.28 ^cd^	13.35 ± 0.18 ^e^	15.86 ± 0.06 ^f^
*C. farreri*	39.61	64.58 ± 0.49 ^a^	6.58 ± 0.34 ^ab^	10.81 ± 0.02 ^f^	13.09 ± 1.41 ^g^
*S. subcrenata*	31.43	55.92 ± 0.76 ^c^	4.41 ± 0.36 ^c^	14.29 ± 0.47 ^de^	16.13 ± 0.66 ^f^
*M. veneriformis*	25.18	47.59 ± 1.06 ^e^	3.35 ± 0.12 ^cd^	19.92 ± 0.25 ^bc^	28.50 ± 0.12 ^b^
*M. meretrix*	11.93	51.01 ± 0.81 ^d^	2.68 ± 0.27 ^de^	19.41 ± 0.02 ^bc^	21.81 ± 0.30 ^d^
*S. constricta*	42.83	52.43 ± 0.47 ^d^	6.78 ± 0.33 ^ab^	25.41 ± 0.68 ^a^	15.49 ± 0.02 ^f^

Different letters within a column indicate significant differences (*p* < 0.05).

**Table 2:** Amino acid contents in 13 shellfish species (g/100 g on a dry basis, *n* = 3).

	*M. chinensis*	*C. sinensis*	*C. gigas*	*S. broughtonii*	*R. philippinarum*	*M. galloprovincialis*	*C. californiense*	*S. strictus*	*C. farreri*	*S. subcrenata*	*M. veneriformis*	*M. meretrix*	*S. constricta*
Asp	4.73 ± 0.01 ^e^	3.23 ± 0.00 ^l^	3.58 ± 0.01 ^i^	5.31 ± 0.01 ^a^	5.21 ± 0.00 ^b^	4.23 ± 0.01 ^g^	4.06 ± 0.00 ^h^	5.00 ± 0.00 ^c^	4.60 ± 0.00 ^f^	5.24 ± 0.00 ^b^	3.35 ± 0.04 ^k^	4.90 ± 0.00 ^d^	3.50 ± 0.00 ^j^
Thr	2.23 ± 0.01 ^e^	1.46 ± 0.00 ^k^	1.64 ± 0.01 ^i^	2.41 ± 0.01 ^b^	2.24 ± 0.00 ^d^	2.10 ± 0.01 ^f^	2.07 ± 0.00 ^g^	2.56 ± 0.00 ^a^	2.24 ± 0.01 ^de^	2.28 ± 0.00 ^c^	1.56 ± 0.01 ^j^	2.22 ± 0.00 ^e^	1.86 ± 0.00 ^h^
Ser	2.34 ± 0.00 ^b^	1.52 ± 0.00 ^k^	1.71 ± 0.01 ^j^	2.32 ± 0.01 ^c^	2.29 ± 0.00 ^d^	2.16 ± 0.01 ^g^	2.07 ± 0.00 ^h^	2.48 ± 0.00 ^a^	2.17 ± 0.00 ^f^	2.20 ± 0.00 ^e^	1.53 ± 0.02 ^k^	2.17 ± 0.00 ^f^	1.97 ± 0.00 ^i^
Glu	6.92 ± 0.01 ^f^	4.38 ± 0.00 ^m^	4.92 ± 0.00 ^k^	8.09 ± 0.01 ^a^	7.02 ± 0.00 ^e^	5.88 ± 0.02 ^h^	5.56 ± 0.01 ^i^	7.82 ± 0.00 ^b^	6.55 ± 0.02 ^g^	7.09 ± 0.00 ^d^	4.84 ± 0.02 ^l^	7.19 ± 0.00 ^c^	5.06 ± 0.00 ^j^
Pro	1.88 ± 0.07 ^cde^	1.34 ± 0.03 ^h^	1.91 ± 0.02 ^cd^	1.95 ± 0.03 ^cd^	1.92 ± 0.01 ^cd^	2.04 ± 0.01 ^ab^	1.61 ± 0.00 ^f^	2.11 ± 0.01 ^a^	1.88 ± 0.06 ^de^	1.95 ± 0.02 ^bcd^	1.47 ± 0.06 ^g^	1.96 ± 0.00 ^bc^	1.82 ± 0.02 ^e^
Gly	4.74 ± 0.00 ^c^	2.02 ± 0.00 ^l^	2.57 ± 0.00 ^j^	3.09 ± 0.01 ^h^	3.58 ± 0.00 ^f^	3.93 ± 0.02 ^e^	4.02 ± 0.00 ^d^	5.36 ± 0.00 ^b^	7.00 ± 0.03 ^a^	2.76 ± 0.00 ^i^	2.39 ± 0.01 ^k^	3.32 ± 0.00 ^g^	2.54 ± 0.01 ^j^
Ala	3.25 ± 0.00 ^d^	2.27 ± 0.00 ^l^	2.03 ± 0.00 ^m^	2.81 ± 0.01 ^g^	3.19 ± 0.00 ^e^	2.37 ± 0.03 ^j^	2.31 ± 0.00 ^k^	3.30 ± 0.00 ^c^	2.52 ± 0.04 ^i^	2.89 ± 0.01 ^f^	4.01 ± 0.01 ^b^	2.71 ± 0.00 ^h^	5.23 ± 0.02 ^a^
Cys	0.20 ± 0.00 ^de^	0.16 ± 0.00 ^f^	0.18 ± 0.00 ^ef^	0.15 ± 0.00 ^f^	0.31 ± 0.00 ^a^	0.28 ± 0.03 ^ab^	0.23 ± 0.00 ^cd^	0.32 ± 0.00 ^a^	0.22 ± 0.04 ^d^	0.27 ± 0.00 ^b^	0.18 ± 0.00 ^ef^	0.20 ± 0.00 ^de^	0.26 ± 0.02 ^bc^
Val	2.00 ± 0.01 ^d^	1.33 ± 0.00 ^h^	1.58 ± 0.00 ^f^	2.13 ± 0.01 ^c^	2.12 ± 0.00 ^c^	1.80 ± 0.07 ^e^	1.82 ± 0.00 ^e^	2.35 ± 0.00 ^a^	2.01 ± 0.10 ^d^	2.24 ± 0.00 ^b^	1.43 ± 0.00 ^g^	2.13 ± 0.00 ^c^	1.63 ± 0.04 ^f^
Met	1.03 ± 0.03 ^ab^	0.56 ± 0.00 ^d^	0.87 ± 0.00 ^c^	0.55 ± 0.01 ^d^	1.07 ± 0.00 ^a^	0.86 ± 0.13 ^c^	0.85 ± 0.00 ^c^	1.10 ± 0.00 ^a^	0.92 ± 0.09 ^bc^	0.94 ± 0.00 ^bc^	0.60 ± 0.01 ^d^	0.88 ± 0.00 ^c^	0.83 ± 0.07 ^c^
Ile	2.10 ± 0.02 ^a^	1.22 ± 0.00 ^d^	1.55 ± 0.00 ^c^	2.18 ± 0.00 ^a^	2.12 ± 0.00 ^a^	1.76 ± 0.15 ^c^	1.82 ± 0.00 ^bc^	2.23 ± 0.00 ^a^	1.94 ± 0.11 ^b^	2.22 ± 0.00 ^a^	1.50 ± 0.00 ^c^	1.91 ± 0.00 ^bc^	1.53 ± 0.14 ^c^
Leu	3.46 ± 0.02 ^c^	2.07 ± 0.00 ^i^	2.47 ± 0.00 ^h^	3.85 ± 0.00 ^a^	3.41 ± 0.00 ^c^	2.84 ± 0.10 ^f^	2.98 ± 0.00 ^e^	3.88 ± 0.00 ^a^	3.27 ± 0.13 ^d^	3.71 ± 0.01 ^b^	2.38 ± 0.00 ^h^	3.34 ± 0.00 ^cd^	2.61 ± 0.09 ^g^
Tyr	1.69 ± 0.01 ^c^	1.14 ± 0.00 ^f^	0.99 ± 0.00 ^g^	1.75 ± 0.00 ^bc^	1.87 ± 0.00 ^a^	1.72 ± 0.07 ^c^	1.54 ± 0.00 ^d^	1.81 ± 0.00 ^ab^	0.04 ± 0.05 ^i^	1.50 ± 0.01 ^d^	0.51 ± 0.00 ^h^	1.54 ± 0.00 ^d^	1.35 ± 0.06 ^e^
Phe	2.56 ± 0.00 ^c^	1.48 ± 0.00 ^i^	2.06 ± 0.01 ^e^	3.48 ± 0.01 ^a^	1.96 ± 0.00 ^f^	2.28 ± 0.05 ^d^	1.85 ± 0.07 ^g^	3.13 ± 0.00 ^b^	2.30 ± 0.01 ^d^	3.14 ± 0.02 ^b^	1.55 ± 0.01 ^h^	2.50 ± 0.01 ^c^	1.82 ± 0.01 ^g^
Lys	3.51 ± 0.00 ^d^	2.23 ± 0.00 ^i^	2.55 ± 0.04 ^g^	3.79 ± 0.02 ^c^	3.55 ± 0.00 ^d^	3.37 ± 0.00 ^e^	3.14 ± 0.09 ^f^	4.20 ± 0.00 ^a^	3.50 ± 0.01 ^d^	3.57 ± 0.05 ^d^	2.43 ± 0.02 ^h^	3.89 ± 0.03 ^b^	2.59 ± 0.01 ^g^
His	1.11 ± 0.00 ^a^	1.13 ± 0.00 ^a^	0.90 ± 0.10 ^bcd^	1.11 ± 0.08 ^a^	1.05 ± 0.00 ^ab^	1.04 ± 0.00 ^ab^	0.90 ± 0.07 ^bcd^	1.20 ± 0.01 ^a^	0.93 ± 0.01 ^bc^	1.13 ± 0.14 ^a^	0.84 ± 0.12 ^cd^	1.16 ± 0.11 ^a^	0.73 ± 0.00 ^d^
Arg	4.21 ± 0.02 ^b^	2.25 ± 0.00 ^k^	2.46 ± 0.03 ^j^	4.23 ± 0.01 ^b^	3.59 ± 0.01 ^f^	3.09 ± 0.01 ^g^	3.76 ± 0.01 ^cd^	4.84 ± 0.00 ^a^	3.80 ± 0.03 ^c^	3.73 ± 0.01 ^de^	2.56 ± 0.01 ^i^	3.73 ± 0.00 ^e^	2.61 ± 0.01 ^h^
Trp	0.78 ± 0.04 ^a^	0.23 ± 0.00 ^e^	0.27 ± 0.01 ^de^	0.74 ± 0.01 ^a^	0.41 ± 0.00 ^cd^	0.79 ± 0.03 ^a^	0.74 ± 0.00 ^a^	0.41 ± 0.00 ^cd^	0.44 ± 0.00 ^c^	0.30 ± 0.01 ^de^	0.34 ± 0.01 ^cde^	0.33 ± 0.01 ^cde^	0.59 ± 0.20 ^b^
EAA	17.67 ± 0.03 ^d^	10.57 ± 0.01 ^k^	13.00 ± 0.07 ^i^	19.13 ± 0.03 ^b^	16.89 ± 0.00 ^ef^	15.79 ± 0.54 ^g^	15.26 ± 0.15 ^h^	19.86 ± 0.01 ^a^	16.62 ± 0.42 ^f^	18.41 ± 0.10 ^c^	11.79 ± 0.01 ^j^	17.20 ± 0.05 ^de^	13.45 ± 0.14 ^i^
NEAA	31.08 ± 0.07 ^b^	19.45 ± 0.03 ^k^	21.27 ± 0.13 ^j^	30.81 ± 0.08 ^b^	30.03 ± 0.01 ^c^	26.74 ± 0.19 ^f^	26.07 ± 0.05 ^g^	34.25 ± 0.01 ^a^	29.69 ± 0.26 ^d^	28.78 ± 0.19 ^e^	21.69 ± 0.09 ^i^	28.88 ± 0.10 ^e^	25.07 ± 0.10 ^h^
TAA	48.75 ± 0.03 ^c^	30.03 ± 0.03 ^l^	34.27 ± 0.20 ^j^	49.94 ± 0.11 ^b^	46.92 ± 0.01 ^de^	42.53 ± 0.73 ^g^	41.33 ± 0.20 ^h^	54.11 ± 0.02 ^a^	46.31 ± 0.68 ^ef^	47.18 ± 0.28 ^d^	33.48 ± 0.10 ^k^	46.08 ± 0.15 ^f^	38.53 ± 0.24 ^i^
EAA/TAA (%)	36.24 ± 0.09 ^ef^	35.21 ± 0.05 ^g^	37.93 ± 0.02 ^b^	38.30 ± 0.02 ^b^	35.99 ± 0.00 ^f^	37.12 ± 0.64 ^cd^	36.93 ± 0.19 ^cd^	36.71 ± 0.00 ^de^	35.88 ± 0.38 ^f^	39.02 ± 0.03 ^a^	35.22 ± 0.08 ^g^	37.32 ± 0.01 ^c^	34.92 ± 0.15 ^g^
EAA/NEAA (%)	56.84 ± 0.23 ^ef^	54.35 ± 0.12 ^g^	61.11 ± 0.06 ^b^	62.08 ± 0.04 ^b^	56.23 ± 0.00 ^f^	59.04 ± 1.62 ^cd^	58.55 ± 0.47 ^cd^	58.00 ± 0.00 ^de^	55.96 ± 0.94 ^f^	63.98 ± 0.07 ^a^	54.36 ± 0.19 ^g^	59.54 ± 0.02 ^c^	53.66 ± 0.35 ^g^

Different letters within a row indicate significant differences (*p* < 0.05); essential amino acid (EAA): Thr, Val, Met, Ile, Leu, Phe, Lys, Trp; nonessential amino acid (NEAA): Asp, Ser, Glu, Pro, Gly, Ala, Cys, Tyr, His, Arg; total amino acid (TAA): the sum of 18 amino acids.

**Table 3:** Amino acid scores of 13 shellfish species.

	FAO/WHO	*M. chinensis*	*C. sinensis*	*C. gigas*	*S. broughtonii*	*R. philippinarum*	*M. galloprovincialis*	*C. californiense*	*S. strictus*	*C. farreri*	*S. subcrenata*	*M. veneriformis*	*M. meretrix*	*S. constricta*
Ile	40	0.81	0.74	0.85	0.89	0.92	0.71	0.71	0.87	0.75	0.99	0.79	0.94	0.73
Leu	70	0.77	0.72	0.77	0.90	0.85	0.66	0.66	0.86	7.36	0.95	0.71	0.94	0.71
Lys	55	0.99	0.98	1.01	1.13	1.12	0.99	0.89	1.19	0.99	1.16	0.93	1.39	0.90
Met + Cys	35	0.55 *	0.50 *	0.66 **	0.33 *	0.68 *	0.53 *	0.48 *	0.63 *	0.51 *	0.62 **	0.47 *	0.60 *	0.60 *
Phe + Tyr	60	1.10	1.06	1.11	1.43	1.11	1.08	0.88	1.28	0.60 **	1.39	0.72	1.32	1.01
Thr	40	0.86	0.88	0.89	0.99	0.98	0.85	0.80	1.00	0.87	1.02	0.82	1.09	0.89
Val	50	0.62 **	0.64	0.69	0.70 **	0.74	0.58 **	0.57 **	0.73	0.62	0.80	0.60 **	0.83	0.62 **
Trp	10	1.21	0.57 **	0.60 *	1.21	0.72 **	1.27	1.15	0.64 **	0.68	0.53 *	0.71	0.64 **	1.12

* The first limiting amino acid; ** The second limiting amino acid.

**Table 4:** Fatty acids profiles of 13 shellfish species (% of total fatty acids, *n* = 3).

	*M. chinensis*	*C. sinensis*	*C. gigas*	*S. broughtonii*	*R. philippinarum*	*M. galloprovincialis*	*C. californiense*	*S. strictus*	*C. farreri*	*S. subcrenata*	*M. veneriformis*	*M. meretrix*	*S. constricta*
C10:0	0.58 ± 0.01 ^b^	0.99 ± 0.02 ^a^	0.15 ± 0.04 ^f^	-	0.32 ± 0.04 ^de^	0.37 ± 0.05 ^de^	-	0.29 ± 0.00 ^e^	-	0.34 ± 0.02 ^de^	0.49 ± 0.04 ^bc^	1.01 ± 0.02 ^a^	0.41 ± 0.04 ^cd^
C14:0	3.30 ± 0.03 ^f^	2.62 ± 0.16 ^g^	4.21 ± 0.12 ^d^	4.03 ± 0.05 ^de^	2.02 ± 0.01 ^h^	1.91 ± 0.04 ^h^	3.77 ± 0.12 ^e^	5.99 ± 0.05 ^b^	2.87 ± 0.05 ^g^	5.29 ± 0.24 ^c^	3.97 ± 0.08 ^de^	1.24 ± 0.07 ^i^	7.73 ± 0.08 ^a^
C15:0	0.79 ± 0.11 ^bc^	0.87 ± 0.07 ^b^	1.07 ± 0.10 ^a^	0.36 ± 0.00 ^e^	0.76 ± 0.00 ^bcd^	0.71 ± 0.03 ^bcd^	0.74 ± 0.07 ^bcd^	0.64 ± 0.03 ^cd^	0.73 ± 0.03 ^bcd^	0.39 ± 0.06 ^e^	0.84 ± 0.07 ^bc^	0.55 ± 0.04 ^de^	1.11 ± 0.05 ^a^
C16:0	20.29 ± 0.11 ^fg^	25.28 ± 0.25 ^c^	29.49 ± 0.22 ^a^	19.94 ± 0.51 ^g^	21.20 ± 0.07 ^def^	22.22 ± 0.20 ^d^	20.50 ± 0.22 ^efg^	19.91 ± 0.11 ^g^	21.58 ± 0.18 ^de^	24.73 ± 0.86 ^c^	21.12 ± 0.20 ^ef^	25.17 ± 0.07 ^c^	27.41 ± 0.13 ^b^
C17:0	1.76 ± 0.06 ^ef^	3.45 ± 0.08 ^a^	2.78 ± 0.02 ^b^	2.14 ± 0.08 ^d^	2.90 ± 0.06 ^b^	3.33 ± 0.05 ^a^	1.45 ± 0.05 ^gh^	1.98 ± 0.04 ^de^	1.71 ± 0.03 ^efg^	2.39 ± 0.08 ^c^	1.30 ± 0.15 ^h^	3.47 ± 0.16 ^a^	1.68 ± 0.08 ^fg^
C18:0	8.08 ± 0.06 ^d^	8.53 ± 0.07 ^c^	6.78 ± 0.03 ^f^	7.46 ± 0.24 ^e^	10.23 ± 0.06 ^a^	6.01 ± 0.06 ^g^	8.91 ± 0.12 ^c^	7.67 ± 0.12 ^de^	6.85 ± 0.02 ^f^	9.36 ± 0.30 ^b^	8.01 ± 0.09 ^d^	9.47 ± 0.15 ^b^	10.01 ± 0.08 ^a^
C20:0	-	-	0.16 ± 0.01 ^a^	-	-	-	0.21 ± 0.02 ^a^	-	-	-	-	-	-
C24:0	-	2.44 ± 0.10 ^a^	-	0.81 ± 0.03 ^d^	2.39 ± 0.03 ^ab^	-	1.19 ± 0.09 ^c^	2.24 ± 0.06 ^ab^	-	1.25 ± 0.05 ^c^	-	2.14 ± 0.12 ^b^	-
SFA	34.80 ± 0.14 ^fg^	44.17 ± 0.03 ^bc^	44.65 ± 0.18 ^b^	34.75 ± 0.27 ^fg^	39.81 ± 0.12 ^d^	34.56 ± 0.03 ^fg^	36.77 ± 0.11 ^e^	38.70 ± 0.13 ^d^	33.74 ± 0.04 ^g^	43.75 ± 1.60 ^bc^	35.74 ± 0.09 ^ef^	43.05 ± 0.05 ^c^	48.35 ± 0.03 ^a^
C14:1	-	-	-	-	-	-	-	0.36 ± 0.06 ^a^	-	0.17 ± 0.02 ^a^	-	-	-
C16:1	7.68 ± 0.02 ^bcd^	7.51 ± 0.12 ^bcd^	3.54 ± 0.02 ^ef^	9.19 ± 0.12 ^bc^	7.59 ± 0.13 ^bcd^	5.38 ± 0.01 ^de^	7.43 ± 0.13 ^bcd^	6.85 ± 0.12 ^cd^	9.12 ± 0.18 ^bc^	10.04 ± 2.83 ^b^	9.83 ± 0.09 ^bc^	2.36 ± 0.18 ^f^	28.94 ± 0.12 ^a^
C18:1 n9 t	-	0.79 ± 0.12	-	-	-	-	-	-	-	-	-	-	-
C18:1 n9 c	3.94 ± 0.11 ^e^	5.41 ± 0.07 ^b^	4.54 ± 0.14 ^d^	2.93 ± 0.01 ^g^	3.47 ± 0.06 ^f^	2.42 ± 0.02 ^h^	1.42 ± 0.07 ^i^	4.88 ± 0.08 ^c^	2.69 ± 0.02 ^gh^	10.39 ± 0.21 ^a^	2.88 ± 0.11 ^g^	3.47 ± 0.07 ^f^	1.60 ± 0.05 ^i^
C20:1	3.36 ± 0.03 ^d^	0.47 ± 0.06 ^i^	0.77 ± 0.06 ^h^	1.21 ± 0.03 ^g^	2.37 ± 0.15 ^ef^	4.80 ± 0.04 ^b^	2.38 ± 0.03 ^ef^	6.50 ± 0.04 ^a^	0.61 ± 0.06 ^hi^	2.11 ± 0.18 ^f^	3.81 ± 0.13 ^c^	2.54 ± 0.06 ^e^	1.15 ± 0.01 ^g^
MUFA	14.97 ± 0.10 ^de^	14.18 ± 0.14 ^ef^	8.85 ± 0.22 ^h^	13.32 ± 0.14 ^efg^	13.43 ± 0.04 ^efg^	12.59 ± 0.04 ^efg^	11.23 ± 0.09 ^g^	18.59 ± 0.10 ^c^	12.41 ± 0.15 ^fg^	22.72 ± 2.44 ^b^	16.52 ± 0.34 ^cd^	8.37 ± 0.05 ^h^	31.70 ± 0.08 ^a^
C18:2 n6 c	0.42 ± 0.07 ^e^	1.20 ± 0.10 ^bc^	1.42 ± 0.09 ^b^	1.77 ± 0.03 ^a^	-	1.32 ± 0.03 ^bc^	0.39 ± 0.06 ^e^	-	1.24 ± 0.05 ^bc^	1.10 ± 0.02 ^c^	0.44 ± 0.06 ^e^	0.74 ± 0.10 ^d^	-
C18:3 n3	0.25 ± 0.01 ^g^	1.93 ± 0.00 ^b^	1.06 ± 0.07 ^d^	1.47 ± 0.02 ^c^	-	1.60 ± 0.07 ^c^	0.60 ± 0.05 ^f^	-	1.63 ± 0.03 ^c^	0.37 ± 0.02 ^g^	0.90 ± 0.04 ^e^	3.10 ± 0.09 ^a^	-
C20:2	1.69 ± 0.11 ^b^	1.25 ± 0.21 ^c^	-	-	1.81 ± 0.01 ^b^	0.37 ± 0.01 ^d^	1.13 ± 0.04 ^c^	1.14 ± 0.03 ^c^	0.25 ± 0.00 ^d^	-	1.87 ± 0.02 ^b^	3.41 ± 0.11 ^a^	-
C20:3 n6	-	-	-	-	-	-	-	0.31 ± 0.02	-	-	-	-	-
C20:4 n6	5.15 ± 0.06 ^c^	6.65 ± 0.04 ^a^	2.04 ± 0.09 ^hi^	1.83 ± 0.01 ^i^	4.23 ± 0.13 ^d^	5.15 ± 0.11 ^c^	1.50 ± 0.10 ^j^	3.37 ± 0.03 ^f^	1.95 ± 0.19 ^i^	2.52 ± 0.10 ^g^	2.31 ± 0.09 ^gh^	6.13 ± 0.03 ^b^	3.69 ± 0.04 ^e^
C20:3 n3	-	0.58 ± 0.02 ^a^	-	-	-	-	-	-	-	-	-	0.70 ± 0.00 ^a^	-
C20:5 n3 (EPA)	20.11 ± 0.09 ^e^	14.69 ± 0.23 ^g^	23.10 ± 0.18 ^d^	29.10 ± 0.17 ^a^	17.42 ± 0.25 ^f^	17.81 ± 0.13 ^f^	25.29 ± 0.17 ^c^	19.37 ± 0.07 ^e^	27.26 ± 0.02 ^b^	19.85 ± 0.51 ^e^	25.25 ± 0.31 ^c^	11.45 ± 0.17 ^h^	-
C22:6 n3 (DHA)	22.61 ± 0.09 ^c^	15.36 ± 0.19 ^i^	18.87 ± 0.15 ^e^	17.76 ± 0.07 ^f^	23.30 ± 0.02 ^b^	26.59 ± 0.19 ^a^	23.08 ± 0.09 ^b^	18.51 ± 0.00 ^e^	21.51 ± 0.01 ^d^	9.70 ± 0.22 ^j^	16.97 ± 0.09 ^g^	23.06 ± 0.09 ^b^	16.26 ± 0.01 ^h^
EPA + DHA	42.72 ± 0.00 ^d^	30.04 ± 0.04 ^h^	41.97 ± 0.03 ^d^	46.86 ± 0.10 ^b^	40.72 ± 0.27 ^e^	44.40 ± 0.06 ^c^	48.37 ± 0.08 ^a^	37.89 ± 0.07 ^f^	48.77 ± 0.03 ^a^	29.55 ± 0.73 ^h^	42.23 ± 0.22 ^d^	34.51 ± 0.09 ^g^	16.26 ± 0.01 ^i^
PUFA	50.23 ± 0.03 ^d^	41.65 ± 0.17 ^i^	46.50 ± 0.04 ^g^	51.93 ± 0.12 ^c^	46.77 ± 0.16 ^g^	52.85 ± 0.07 ^b^	52.00 ± 0.02 ^c^	42.71 ± 0.03 ^h^	53.84 ± 0.19 ^a^	33.53 ± 0.84 ^j^	47.74 ± 0.26 ^f^	48.58 ± 0.00 ^e^	19.95 ± 0.05 ^k^
n-3/n-6	7.72 ± 0.18 ^e^	4.15 ± 0.03 ^g^	12.42 ± 0.03 ^c^	13.44 ± 0.13 ^c^	9.62 ± 0.35 ^d^	7.11 ± 0.08 ^e^	25.82 ± 0.58 ^a^	10.28 ± 0.04 ^d^	15.82 ± 1.23 ^b^	8.29 ± 0.01 ^e^	15.73 ± 0.22 ^b^	5.58 ± 0.08 ^f^	4.40 ± 0.04 ^fg^

Different letters within a row indicate significant differences (*p* < 0.05); - not detected; saturated fatty acid (SFA); monounsaturated fatty acids (MUFA); polyunsaturated fatty acid (PUFA).

**Table 5:** Free amino acids contents in 13 shellfish species (g/100 g on dry basis, *n* = 3).

	*M. chinensis*	*C. sinensis*	*C. gigas*	*S. broughtonii*	*R. philippinarum*	*M. galloprovincialis*	*C. californiense*	*S. strictus*	*C. farreri*	*S. subcrenata*	*M. veneriformis*	*M. meretrix*	*S. constricta*
Asp *	0.16 ± 0.00 ^i^	0.17 ± 0.00 ^h^	0.41 ± 0.01 ^c^	0.36 ± 0.00 ^d^	0.45 ± 0.00 ^a^	0.34 ± 0.00 ^e^	0.15 ± 0.00 ^j^	0.22 ± 0.00 ^f^	0.10 ± 0.00 ^k^	0.45 ± 0.00 ^a^	0.20 ± 0.00 ^g^	0.44 ± 0.00 ^b^	0.22 ± 0.00 ^f^
Thr ^#^	0.18 ± 0.00 ^cd^	0.17 ± 0.00 ^cde^	0.18 ± 0.00 ^cd^	0.21 ± 0.00 ^cd^	0.32 ± 0.00 ^b^	0.24 ± 0.01 ^bc^	0.32 ± 0.00 ^b^	0.09 ± 0.12 ^e^	0.21 ± 0.00 ^cd^	0.13 ± 0.00 ^de^	0.18 ± 0.00 ^cd^	0.19 ± 0.05 ^cd^	0.45 ± 0.00 ^a^
Ser ^#^	0.15 ± 0.00 ^e^	0.13 ± 0.00 ^f^	0.30 ± 0.00 ^b^	0.11 ± 0.01 ^g^	0.23 ± 0.00 ^c^	0.18 ± 0.00 ^d^	0.14 ± 0.00 ^ef^	0.18 ± 0.00 ^d^	0.18 ± 0.00 ^d^	0.08 ± 0.00 ^h^	0.12 ± 0.00 ^g^	0.13 ± 0.00 ^f^	0.8 ± 0.01 ^a^
Glu *	1.16 ± 0.00 ^d^	0.92 ± 0.00 ^f^	0.86 ± 0.01 ^gh^	1.30 ± 0.03 ^b^	1.31 ± 0.00 ^b^	1.03 ± 0.01 ^e^	0.92 ± 0.00 ^f^	1.26 ± 0.03 ^c^	0.85 ± 0.00 ^hi^	0.90 ± 0.01 ^fg^	1.29 ± 0.01 ^bc^	1.61 ± 0.00 ^a^	0.83 ± 0.00 ^i^
Pro ^#^	-	-	1.12 ± 0.20 ^a^	0.01 ± 0.02 ^d^	-	-	-	-	-	0.44 ± 0.07 ^c^	-	-	0.93 ± 0.19 ^b^
Gly ^#^	4.43 ± 0.00 ^c^	0.78 ± 0.00 ^l^	1.27 ± 0.00 ^j^	1.09 ± 0.01 ^k^	2.27 ± 0.00 ^e^	1.62 ± 0.00 ^g^	3.71 ± 0.01 ^d^	4.48 ± 0.01 ^b^	8.40 ± 0.03 ^a^	0.50 ± 0.00 ^m^	1.67 ± 0.00 ^f^	1.45 ± 0.00 ^h^	1.30 ± 0.00 ^i^
Ala ^#^	1.60 ± 0.00 ^c^	1.56 ± 0.00 ^d^	0.72 ± 0.00 ^f^	0.27 ± 0.01 ^k^	1.56 ± 0.00 ^d^	0.51 ± 0.00 ^j^	0.69 ± 0.00 ^g^	0.99 ± 0.00 ^e^	0.56 ± 0.00 ^i^	0.57 ± 0.00 ^h^	4.78 ± 0.02 ^b^	0.54 ± 0.00 ^i^	6.67 ± 0.02 ^a^
Cys	0.03 ± 0.00 ^de^	0.06 ± 0.00 ^b^	0.05 ± 0.00 ^bc^	0.03 ± 0.01 ^de^	0.06 ± 0.00 ^b^	0.03 ± 0.01 ^e^	0.04 ± 0.00 ^cd^	-	0.03 ± 0.00 ^de^	-	0.03 ± 0.00 ^de^	-	0.08 ± 0.00 ^a^
Val ^▪^	0.10 ± 0.01 ^ef^	0.09 ± 0.00 ^f^	0.13 ± 0.00 ^d^	0.09 ± 0.02 ^f^	0.15 ± 0.00 ^c^	0.12 ± 0.01 ^de^	0.24 ± 0.00 ^b^	0.12 ± 0.00 ^de^	0.28 ± 0.00 ^a^	0.07 ± 0.00 ^g^	0.13 ± 0.00 ^d^	0.12 ± 0.00 ^de^	0.23 ± 0.00 ^b^
Met ^▪^	0.04 ± 0.01 ^c^	0.04 ± 0.00 ^c^	0.11 ± 0.00 ^b^	0.04 ± 0.01 ^c^	0.16 ± 0.00 ^a^	0.06 ± 0.02 ^c^	0.11 ± 0.00 ^b^	0.07 ± 0.00 ^c^	0.06 ± 0.00 ^c^	0.05 ± 0.00 ^c^	0.06 ± 0.01 ^c^	0.05 ± 0.00 ^c^	0.18 ± 0.01 ^a^
Ile ^▪^	0.05 ± 0.01 ^de^	0.07 ± 0.00 ^cde^	0.04 ± 0.00.^e^	0.06 ± 0.02 ^cde^	0.10 ± 0.00 ^bc^	0.10 ± 0.04 ^bcd^	0.15 ± 0.00 ^a^	0.05 ± 0.00 ^de^	0.06 ± 0.00 ^cde^	0.04 ± 0.00 ^e^	0.07 ± 0.01 ^cde^	0.07 ± 0.00 ^cde^	0.12 ± 0.01 ^ab^
Leu ^▪^	0.10 ± 0.06 ^def^	0.15 ± 0.00 ^bcdef^	0.08 ± 0.01 ^ef^	0.06 ± 0.00 ^f^	0.20 ± 0.00 ^bc^	0.18 ± 0.04 ^bcd^	0.29 ± 0.00 ^a^	0.13 ± 0.00 ^bcdef^	0.16 ± 0.00 ^bcde^	0.12 ± 0.00 ^cdef^	0.13 ± 0.04 ^bcdef^	0.14 ± 0.00 ^bcdef^	0.22 ± 0.01 ^b^
Tyr ^▪^	-	0.17 ± 0.00 ^ab^	-	-	0.15 ± 0.01 ^ab^	0.11 ± 0.15 ^ab^	0.24 ± 0.00 ^a^	0.16 ± 0.01 ^ab^	0.17 ± 0.01 ^ab^	0.13 ± 0.01 ^ab^	0.07 ± 0.10 ^ab^	0.12 ± 0.00 ^ab^	0.24 ± 0.01 ^a^
Phe ^▪^	0.35 ± 0.06 ^e^	0.24 ± 0.00 ^f^	0.75 ± 0.00 ^c^	2.30 ± 0.03 ^a^	0.22 ± 0.00 ^f^	0.53 ± 0.01 ^d^	0.77 ± 0.02 ^c^	0.32 ± 0.00 ^e^	0.24 ± 0.00 ^f^	1.24 ± 0.01 ^b^	0.34 ± 0.00 ^e^	0.23 ± 0.00 ^f^	0.51 ± 0.00 ^d^
Lys ^▪^	0.17 ± 0.07 ^cd^	0.08 ± 0.00 ^d^	0.16 ± 0.00 ^cd^	0.18 ± 0.08 ^cd^	0.21 ± 0.00 ^bc^	0.37 ± 0.01 ^a^	0.31 ± 0.00 ^ab^	0.10 ± 0.00 ^cd^	0.13 ± 0.00 ^cd^	0.10 ± 0.00 ^cd^	0.17 ± 0.00 ^cd^	0.17 ± 0.00 ^cd^	0.30 ± 0.00 ^ab^
His ^▪^	0.10 ± 0.01 ^bc^	0.04 ± 0.00 ^e^	0.12 ± 0.00 ^b^	0.13 ± 0.00 ^b^	0.05 ± 0.00 ^de^	0.12 ± 0.02 ^b^	0.12 ± 0.00 ^b^	0.08 ± 0.00 ^cd^	0.05 ± 0.00 ^de^	0.06 ± 0.00 ^de^	0.06 ± 0.00 ^de^	0.07 ± 0.00 ^de^	0.24 ± 0.02 ^a^
Arg ^#^	2.22 ± 0.00 ^b^	0.58 ± 0.01 ^k^	0.65 ± 0.00 ^j^	1.27 ± 0.00 ^e^	1.04 ± 0.00 ^g^	0.36 ± 0.02 ^l^	1.96 ± 0.00 ^c^	2.26 ± 0.00 ^a^	1.79 ± 0.00 ^d^	0.64 ± 0.00 ^j^	1.11 ± 0.00 ^f^	0.70 ± 0.00 ^i^	0.79 ± 0.00 ^h^
Tau	0.93 ± 0.01 ^d^	0.91 ± 0.01 ^d^	1.04 ± 0.02 ^c^	1.14 ± 0.03 ^b^	1.15 ± 0.02 ^b^	1.11 ± 0.02 ^b^	0.88 ± 0.02 ^d^	0.91 ± 0.01 ^d^	0.56 ± 0.02 ^f^	1.32 ± 0.02 ^a^	0.72 ± 0.01 ^e^	1.01 ± 0.02 ^c^	0.56 ± 0.01 ^f^
UFAAs	1.32 ± 0.00 ^e^	1.09 ± 0.00 ^g^	1.28 ± 0.01 ^f^	1.66 ± 0.03 ^c^	1.76 ± 0.00 ^b^	1.37 ± 0.01 ^e^	1.08 ± 0.00 ^g^	1.48 ± 0.03 ^d^	0.95 ± 0.00 ^h^	1.35 ± 0.01 ^e^	1.49 ± 0.02 ^d^	2.05 ± 0.00 ^a^	1.05 ± 0.00 ^g^
SFAAs	8.58 ± 0.01 ^b^	3.23 ± 0.01 ^g^	4.26 ± 0.21 ^f^	2.95 ± 0.00 ^gh^	5.42 ± 0.01 ^e^	2.90 ± 0.02 ^h^	6.82 ± 0.01 ^d^	8.00 ± 0.11 ^c^	11.14 ± 0.02 ^a^	2.37 ± 0.07 ^i^	7.85 ± 0.02 ^c^	3.02 ± 0.05 ^gh^	10.93 ± 0.22 ^a^
BFAAs	0.92 ± 0.06 ^gh^	0.87 ± 0.01 ^h^	1.40 ± 0.00 ^de^	2.87 ± 0.11 ^a^	1.24 ± 0.01 ^ef^	1.58 ± 0.13 ^d^	2.23 ± 0.02 ^b^	1.02 ± 0.01 ^gh^	1.15 ± 0.01 ^fg^	1.81 ± 0.02 ^c^	1.03 ± 0.14 ^gh^	0.97 ± 0.00 ^gh^	2.03 ± 0.02 ^b^
TFAAs	11.78 ± 0.04 ^c^	6.16 ± 0.00 ^i^	8.03 ± 0.24 ^g^	8.65 ± 0.04 ^f^	9.63 ± 0.03 ^e^	6.98 ± 0.13 ^h^	11.04 ± 0.03 ^d^	11.41 ± 0.15 ^cd^	13.83 ± 0.02 ^b^	6.85 ± 0.12 ^h^	11.12 ± 0.18 ^d^	7.05 ± 0.04 ^h^	14.65 ± 0.24 ^a^

Different letters within a row indicate significant differences (*p* < 0.05); - not detected; * umami free amino acid; ^#^ sweet free amino acid; ^▪^ bitter free amino acid; TFAAs, UFAAs, SFAAs, BFAAs and TFAAs were umami free amino acids (Asp and Glu), sweet free amino acids (Thr, Ser, Pro, Gly, Ala, and Arg), bitter free amino acids (Val, Met, Ile, Leu, Tyr, Phe, Lys, and His), total free amino acids, respectively.

**Table 6:** Free amino acids contents in boiling liquids of 13 shellfish species (g/100 g on dry basis, *n* = 3).

	*M. chinensis*	*C. sinensis*	*C. gigas*	*S. broughtonii*	*R. philippinarum*	*M. galloprovincialis*	*C. californiense*	*S. strictus*	*C. farreri*	*S. subcrenata*	*M. veneriformis*	*M. meretrix*	*S. constricta*
Asp *	0.34 ± 0.00 ^j^	0.20 ± 0.00 ^l^	0.70 ± 0.00 ^c^	0.67 ± 0.00 ^d^	1.19 ± 0.00 ^a^	0.61 ± 0.00 ^e^	0.39 ± 0.00 ^h^	0.43 ± 0.00 ^f^	0.27 ± 0.00 ^k^	1.13 ± 0.00 ^b^	0.42 ± 0.00 ^g^	0.37 ± 0.00 ^i^	0.39 ± 0.00 ^h^
Thr ^#^	0.23 ± 0.00 ^c^	0.22 ± 0.00 ^c^	0.23 ± 0.00 ^c^	0.18 ± 0.00 ^d^	-	0.13 ± 0.00 ^e^	0.68 ± 0.00 ^a^	-	0.31 ± 0.00 ^b^	0.12 ± 0.00 ^f^	0.31 ± 0.00 ^b^	0.06 ± 0.00 ^g^	0.68 ± 0.00 ^a^
Ser ^#^	0.18 ± 0.00 ^g^	0.12 ± 0.00 ^i^	0.25 ± 0.00 ^e^	0.06 ± 0.00 ^l^	0.35 ± 0.00 ^b^	0.15 ± 0.00 ^h^	0.26 ± 0.00 ^d^	0.23 ± 0.00 ^f^	0.31 ± 0.00 ^c^	0.08 ± 0.00 ^k^	0.10 ± 0.00 ^j^	0.09 ± 0.00 ^j^	1.50 ± 0.00 ^a^
Glu *	1.69 ± 0.00 ^e^	1.06 ± 0.00 ^l^	0.96 ± 0.00 ^m^	1.20 ± 0.00 ^k^	2.16 ± 0.00 ^b^	1.50 ± 0.00 ^i^	1.64 ± 0.00 ^f^	1.99 ± 0.00 ^c^	1.52 ± 0.00 ^h^	1.71 ± 0.00 ^d^	2.45 ± 0.00 ^a^	1.49 ± 0.00 ^j^	1.58 ± 0.00 ^g^
Pro ^#^	-	-	1.02 ± 0.04 ^b^	-	-	-	0.50 ± 0.02 ^c^	0.31 ± 0.01 ^e^	-	0.45 ± 0.01 ^d^	0.42 ± 0.01 ^d^	-	1.22 ± 0.01 ^a^
Gly ^#^	7.03 ± 0.01 ^c^	1.00. ± 0.00 ^l^	1.77 ± 0.00 ^i^	0.17 ± 0.00 ^m^	3.92 ± 0.00 ^f^	2.76 ± 0.00 ^h^	6.71 ± 0.00 ^d^	7.78 ± 0.00 ^b^	16.97 ± 0.02 ^a^	1.21 ± 0.00 ^k^	3.60 ± 0.01 ^g^	1.55 ± 0.00 ^j^	4.17 ± 0.00 ^e^
Ala ^#^	2.67 ± 0.00 ^d^	2.27 ± 000 ^e^	0.83 ± 0.00 ^j^	0.45 ± 0.00 ^m^	2.81 ± 0.00 ^c^	0.79 ± 0.00 ^k^	1.57 ± 0.00 ^g^	1.85 ± 0.00 ^f^	0.88 ± 0.00 ^i^	1.27 ± 0.00 ^h^	11.84 ± 0.02 ^b^	0.57 ± 0.00 ^l^	13.31 ± 0.00 ^a^
Cys	-	-	-	-	-	-	-	-	-	-	-	-	-
Val^▪^	0.12 ± 0.00 ^f^	0.08 ± 0.00 ^g^	0.15 ± 0.00 ^e^	0.11 ± 0.00	0.14 ± 0.01 ^e^	0.11 ± 0.00 ^f^	0.53 ± 0.00 ^a^	0.17 ± 0.00 ^d^	0.49 ± 0.00 ^b^	0.11 ± 0.00 ^f^	0.15 ± 0.00 ^e^	0.05 ± 0.00 ^h^	0.37 ± 0.00 ^c^
Met^▪^	0.07 ± 0.00 ^ef^	0.03 ± 0.00 ^g^	0.10 ± 0.00 ^cd^	0.05 ± 0.00 ^fg^	0.07 ± 0.02 ^de^	0.11 ± 0.00 ^c^	0.21 ± 0.00 ^b^	0.08 ± 0.01 ^de^	0.11 ± 0.00 ^c^	0.07 ± 0.00 ^ef^	0.06 ± 0.00 ^ef^	0.02 ± 0.00 ^g^	0.29 ± 0.00 ^a^
Ile^▪^	0.03 ± 0.00 ^e^	0.05 ± 0.00 ^d^	0.06 ± 0.00.^cd^	0.05 ± 0.00 ^cd^	-	0.07 ± 0.00 ^c^	0.36 ± 0.00 ^a^	0.07 ± 0.00 ^c^	0.07 ± 0.00 ^c^	0.05 ± 0.00 ^d^	0.06 ± 0.00 ^cd^	0.02 ± 0.00 ^e^	0.17 ± 0.01 ^b^
Leu^▪^	0.05 ± 0.00 ^h^	0.12 ± 0.00 ^e^	0.15 ± 000 ^d^	0.11 ± 0.00 ^f^	0.16 ± 0.00 ^cd^	0.13 ± 0.00 ^e^	0.58 ± 0.00 ^a^	0.13 ± 0.00 ^e^	0.17 ± 0.00 ^c^	0.12 ± 0.00 ^ef^	0.13 ± 0.00 ^e^	0.08 ± 0.00 ^g^	0.24 ± 0.01 ^b^
Tyr^▪^	-	0.15 ± 0.00 ^d^	0.13 ± 0.00 ^ef^	0.12 ± 0.00 ^f^	0.1 ± 0.00 ^g^	0.21 ± 0.00 ^b^	0.35 ± 0.00 ^a^	0.15 ± 0.00 ^d^	0.17 ± 0.00 ^c^	0.14 ± 0.00 ^de^	0.16 ± 0.00 ^c^	0.08 ± 0.00 ^h^	0.22 ± 0.01 ^b^
Phe ^▪^	-	0.15 ± 0.00 ^d^	0.79 ± 0.01 ^a^	-	0.25 ± 0.02 ^c^	-	-	-	0.51 ± 0.00 ^b^	-	0.51 ± 0.00 ^b^	0.10 ± 0.00 ^e^	0.55 ± 0.04 ^b^
Lys ^▪^	0.15 ± 0.06 ^bcde^	0.06 ± 0.00 ^e^	0.16 ± 0.00 ^bcde^	0.12 ± 0.00 ^cde^	0.26 ± 0.00 ^bc^	0.15 ± 0.00 ^bcde^	0.48 ± 0.00 ^a^	0.22 ± 0.09 ^bcd^	0.23 ± 0.00 ^bcd^	0.18 ± 0.00 ^bcde^	0.27 ± 0.00 ^b^	0.10 ± 0.00 ^de^	0.28 ± 0.09 ^b^
His ^▪^	0.09 ± 0.03 ^cd^	0.04 ± 0.00 ^d^	0.12 ± 0.00.^bc^	0.12 ± 0.00.^bc^	0.07 ± 0.00 ^cd^	0.12 ± 0.00 ^bc^	0.18 ± 0.00 ^ab^	0.14 ± 0.04 ^abc^	0.10 ± 0.00 ^cd^	0.11 ± 0.00.^c^	0.12 ± 0.00 ^bc^	0.04 ± 0.00 ^d^	0.19 ± 0.04 ^a^
Arg ^#^	2.86 ± 0.00 ^c^	0.70 ± 0.00 ^j^	0.67 ± 000 ^k^	0.5 ± 000.^m^	1.50 ± 0.00 ^g^	0.70 ± 0.00 ^i^	3.17 ± 0.01 ^b^	3.85 ± 0.00 ^a^	1.95 ± 0.00 ^e^	1.27 ± 000 ^h^	2.44 ± 0.00 ^d^	0.65 ± 0.00 ^l^	1.79 ± 0.00 ^f^
Tau	1.01 ± 0.01 ^f^	1.07 ± 0.01 ^e^	0.96 ± 0.01 ^g^	1.37 ± 0.00 ^b^	1.24 ± 0.01 ^d^	1.31 ± 0.02 ^c^	0.84 ± 0.01 ^h^	1.02 ± 0.00 ^f^	0.53 ± 0.01 ^j^	1.47 ± 0.01 ^a^	0.85 ± 0.00 ^h^	1.06 ± 0.00 ^e^	0.64 ± 0.01 ^i^
UFAAs	2.03 ± 0.00 ^f^	1.26 ± 0.00 ^l^	1.66 ± 0.00 ^k^	1.87 ± 0.00 ^h^	3.34 ± 0.00 ^a^	2.11 ± 0.00 ^e^	2.03 ± 0.00 ^f^	2.42 ± 0.00 ^d^	1.79 ± 0.00 ^j^	2.84 ± 0.00 ^c^	2.87 ± 0.00 ^b^	1.86 ± 0.00 ^i^	1.97 ± 0.00 ^g^
SFAAs	12.97 ± 0.01 ^e^	4.32 ± 0.00 ^k^	4.76 ± 0.06 ^h^	1.36 ± 0.00 ^m^	8.58 ± 0.00 ^g^	4.54 ± 0.00 ^i^	12.88 ± 0.02 ^f^	14.03 ± 0.02 ^d^	20.42 ± 0.02 ^b^	4.41 ± 0.02 ^j^	18.71 ± 0.02 ^c^	2.93 ± 0.00 ^l^	22.68 ± 0.01 ^a^
BFAAs	0.50 ± 0.08 ^c^	0.68 ± 0.00 ^c^	1.27 ± 0.57 ^bc^	0.68 ± 0.00 ^c^	1.05 ± 0.05 ^bc^	0.90 ± 0.00 ^bc^	2.69 ± 0.00 ^a^	0.96 ± 0.15 ^bc^	1.60 ± 0.36 ^b^	0.78 ± 0.01 ^bc^	1.20 ± 0.36 ^bc^	0.47 ± 0.00 ^c^	2.30 ± 0.20 ^a^
TFAAs	16.51 ± 0.09 ^e^	7.33 ± 0.01 ^i^	8.65 ± 0.49 ^h^	5.28 ± 0.01 ^k^	14.20 ± 0.06 ^f^	8.87 ± 0.02 ^h^	18.44 ± 0.01 ^d^	18.43 ± 0.13 ^d^	24.33 ± 0.33 ^b^	9.49 ± 0.01 ^g^	23.63 ± 0.35 ^c^	6.32 ± 0.01 ^j^	27.59 ± 0.19 ^a^

Different letters within a row indicate significant difference (*p* < 0.05); - not detected; * umami free amino acids (UFAAs); ^#^ sweet free amino acids (SFAAs); ^▪^ bitter free amino acids (BFAAs); total free amino acids (TFAAs).

**Table 7:** Nucleotide contents (mg/100 g on a dry basis, *n* = 3) in 13 shellfish meat and their boiling liquids.

Samples	Nucleotides	GMP	IMP	AMP	CMP	UMP
Shellfish meat	*M. chinensis*	33.45 ± 0.07 ^f^	7.90 ± 0.03 ^c^	-	-	34.48 ± 0.27 ^b^
	*C. sinensis*	31.88 ± 0.13 ^g^	8.18 ± 0.07 ^c^	-	-	7.71 ± 0.06 ^h^
	*C. gigas*	42.38 ± 0.05 ^e^	7.05 ± 0.01 ^d^	-	-	16.99 ± 0.11 ^e^
	*S. broughtonii*	15.56 ± 0.01 ^i^	-	-	1.31 ± 0.03 ^b^	5.51 ± 0.00 ^i^
	*R. philippinarum*	57.56 ± 0.01 ^d^	52.57 ± 0.19 ^b^	1.49 ± 0.00 ^c^	-	44.45 ± 0.03 ^a^
	*M. galloprovincialis*	151.51 ± 0.53 ^a^	-	1.63 ± 0.01 ^c^	-	5.12 ± 0.02 ^i^
	*C. californiense*	99.91 ± 0.17 ^b^	-	-	-	10.08 ± 0.21 ^g^
	*S. strictus*	71.72 ± 0.32 ^c^	-	-	-	11.08 ± 0.03 ^f^
	*C. farreri*	41.80 ± 0.51 ^e^	0.42 ± 0.01 ^f^	-	-	3.83 ± 0.00 ^k^
	*S. subcrenata*	31.86 ± 0.02 ^g^	-	2.65 ± 0.11 ^b^	-	4.51 ± 0.01 ^j^
	*M. veneriformis*	42.52 ± 0.14 ^e^	3.84 ± 0.33 ^e^	4.68 ± 0.17 ^a^	-	28.67 ± 0.46 ^d^
	*M. meretrix*	99.01 ± 0.02 ^b^	63.10 ± 0.17 ^a^	1.37 ± 0.02 ^c^	2.75 ± 0.34 ^a^	31.89 ± 0.01 ^c^
	*S. constricta*	25.69 ± 0.04 ^h^	-	-	-	2.11 ± 0.05 ^l^
Boiling liquids	*M. chinensis*	17.04 ± 0.02 ^h^	7.87 ± 0.13 ^e^	-	-	48.13 ± 0.16 ^g^
	*C. sinensis*	8.51 ± 0.28 ^l^	1.91 ± 0.09 ^i^	-	-	22.72 ± 0.39 ^j^
	*C. gigas*	16.27 ± 0.13 ^i^	3.17 ± 0.04 ^h^	-	6.03 ± 0.06 ^a^	54.97 ± 0.09 ^f^
	*S. broughtonii*	9.28 ± 0.08 ^k^	-	-	5.98 ± 0.41 ^a^	22.62 ± 0.02 ^j^
	*R. philippinarum*	25.28 ± 0.00 ^e^	45.64 ± 0.01 ^a^	-	-	57.67 ± 0.00 ^e^
	*M. galloprovincialis*	28.18 ± 0.06 ^d^	9.83 ± 0.00 ^d^	-	-	61.43 ± 0.42 ^d^
	*C. californiense*	64.02 ± 0.00 ^a^	28.13 ± 0.38 ^b^	-	-	125.87 ± 0.12 ^a^
	*S. strictus*	22.90 ± 0.25 ^f^	3.66 ± 0.14 ^h^	-	-	42.49 ± 0.03 ^h^
	*C. farreri*	47.56 ± 0.05 ^b^	21.47 ± 0.20 ^c^	-	-	96.33 ± 0.24 ^b^
	*S. subcrenata*	19.32 ± 0.01 ^g^	5.18 ± 0.08 ^g^	-	-	48.14 ± 0.28 ^g^
	*M. veneriformis*	28.46 ± 0.06 ^d^	9.72 ± 0.00 ^d^	-	4.63 ± 0.35 ^b^	54.43 ± 0.10 ^f^
	*M. meretrix*	12.44 ± 0.00 ^j^	5.85 ± 0.41 ^f^	-	-	29.59 ± 0.06 ^i^
	*S. constricta*	39.01 ± 0.28 ^c^	4.75 ± 0.02 ^g^	-	-	74.46 ± 0.00 ^c^

Different letters within a column indicate significant difference (*p* < 0.05); - not detected; 5′-monophosphate (GMP); inosine 5′-monophosphate (IMP); adenosine 5′-monophosphate (AMP); 5′-monohydrocytidine disodium (CMP); 5′-monophosphoric acid uridine disodium (UMP).

**Table 9:** Organic acids contents (mg/100 g on a dry basis, *n* = 3) in 13 shellfish meat and their boiling liquids.

Samples	Organic Acids	Lactic Acid	Succinic Acid	Malic Acid	Oxalic Acid	Tartaric Acid	Acetic Acid	Maleic Acid	Fumaric Acid	Total Organic Acids
Shellfish meat	*M. chinensis*	190.59 ± 5.33 ^i^	1364.14 ± 6.67 ^c^	830.64 ± 10.47 ^e^	599.07 ± 15.29 ^a^	2971.66 ± 30.26 ^c^	479.50 ± 7.52 ^d^	0.56 ± 0.09 ^f^	1.37 ± 0.13 ^h^	6437.53 ± 64.91 ^e^
	*C. sinensis*	3.41 ± 0.82 ^j^	1287.02 ± 8.40 ^d^	366.12 ± 7.44 ^i^	98.21 ± 6.67 ^c^	819.12 ± 6.69 ^k^	145.93 ± 2.08 ^g^	0.16 ± 0.03 ^f^	4.36 ± 0.25 ^c^	2724.34 ± 12.69 ^l^
	*C. gigas*	1553.09 ± 13.51 ^d^	-	536.06 ± 7.62 ^g^	32.61 ± 2.28 ^ef^	2004.41 ± 13.20 ^f^	-	45.25 ± 1.34 ^b^	1.54 ± 0.06 ^gh^	4172.95 ± 3.74 ^i^
	*S. broughtonii*	1423.18 ± 9.91 ^e^	1964.32 ± 21.66 ^a^	477.06 ± 5.08 ^h^	19.78 ± 2.67 ^fgh^	5276.14 ± 15.97 ^a^	33.23 ± 2.36 ^h^	26.80 ± 2.42 ^d^	2.43 ± 0.09 ^ef^	9222.96 ± 25.17 ^a^
	*R. philippinarum*	3862.63 ± 4.69 ^a^	405.20 ± 15.25 ^h^	1015.89 ± 7.67 ^c^	169.26 ± 3.39 ^b^	2520.29 ± 16.04 ^d^	829.42 ± 10.61 ^a^	72.40 ± 2.71 ^a^	1.97 ± 0.12 ^fg^	8877.08 ± 20.37 ^b^
	*M. galloprovincialis*	3325.94 ± 8.14 ^b^	345.82 ± 10.37 ^i^	298.92 ± 8.30 ^j^	16.23 ± 2.51 ^fgh^	1090.09 ± 6.93 ^i^	253.78 ± 5.30 ^f^	27.96 ± 0.80 ^d^	1.79 ± 0.05 ^gh^	5360.53 ± 6.21 ^g^
	*C. californiense*	1020.03 ± 13.24 ^f^	1043.69 ± 14.36 ^e^	301.49 ± 5.62 ^j^	14.77 ± 1.15 ^fgh^	1639.86 ± 10.50 ^g^	341.77 ± 3.66 ^e^	3.08 ± 0.05 ^f^	3.12 ± 0.11 ^d^	4367.82 ± 10.16 ^h^
	*S. strictus*	409.24 ± 8.13 ^h^	923.05 ± 9.95 ^g^	2714.89 ± 14.67 ^a^	9.83 ± 1.68 ^gh^	1098.86 ± 10.61 ^i^	552.41 ± 5.75 ^c^	14.45 ± 0.05 ^e^	5.69 ± 0.06 ^b^	5728.43 ± 1.46 ^f^
	*C. farreri*	1572.54 ± 11.36 ^d^	-	208.34 ± 10.03 ^k^	6.36 ± 0.80 ^h^	1024.36 ± 5.82 ^j^	-	38.84 ± 1.16 ^c^	0.47 ± 0.05 ^i^	2850.94 ± 15.89 ^k^
	*S. subcrenata*	651.84 ± 5.77 ^g^	1354.40 ± 13.28 ^c^	285.51 ± 5.40 ^j^	29.01 ± 1.80 ^efg^	4677.03 ± 16.63 ^b^	151.93 ± 1.69 ^g^	15.95 ± 0.45 ^e^	2.73 ± 0.11 ^de^	7168.41 ± 0.08 ^d^
	*M. veneriformis*	1900.04 ± 12.08 ^c^	924.68 ± 26.71 ^g^	650.52 ± 8.76 ^f^	44.61 ± 1.66 ^e^	2325.27 ± 20.45 ^e^	646.14 ± 4.23 ^b^	0.05 ± 0.01	6.34 ± 0.20 ^a^	6497.67 ± 5.68 ^e^
	*M. meretrix*	-	1541.94 ± 36.10 ^b^	875.53 ± 12.08 ^d^	65.94 ± 2.58 ^d^	319.01 ± 5.69 ^l^	255.18 ± 3.06 ^f^	17.81 ± 1.32 ^e^	4.11 ± 0.18 ^c^	3079.50 ± 19.35 ^j^
	*S. constricta*	3327.95 ± 23.41 ^b^	986.63 ± 9.96 ^f^	1140.15 ± 11.36 ^b^	599.07 ± 11.63 ^a^	1211.70 ± 10.33 ^h^	29.77 ± 1.85 ^h^	45.16 ± 1.08 ^b^	2.67 ± 0.36 ^de^	7343.09 ± 47.26 ^c^
Boiling liquids	*M. chinensis*	427.48 ± 12.23 ^h^	1389.83 ± 4.83 ^e^	908.78 ± 5.97 ^h^	400.99 ± 7.22 ^a^	5147.79 ± 19.52 ^d^	991.02 ± 13.25 ^a^	3.91 ± 0.23 ^i^	7.56 ± 0.52 ^f^	9277.36 ± 11.74 ^f^
	*C. sinensis*	361.02 ± 18.90 ^i^	1254.99 ± 16.82 ^f^	644.95 ± 8.83 ^i^	27.76 ± 2.16 ^g^	856.05 ± 13.72 ^l^	128.98 ± 4.56 ^g^	3.70 ± 0.14 ^i^	2.06 ± 0.06 ^h^	3279.52 ± 24.19 ^l^
	*C. gigas*	888.59 ± 14.62 ^e^	338.37 ± 8.61 ^i^	495.23 ± 11.34 ^j^	16.71 ± 1.83 ^h^	2104.76 ± 9.80 ^k^	-	13.35 ± 0.55 ^d^	3.77 ± 0.14 ^g^	3860.77 ± 26.21 ^j^
	*S. broughtonii*	483.05 ± 23.16 ^gh^	734.90 ± 15.39 ^h^	875.22 ± 13.52 ^h^	64.24 ± 2.64 ^e^	8193.76 ± 17.67 ^b^	166.07 ± 4.71 ^f^	7.10 ± 0.06 ^g^	13.88 ± 0.66 ^d^	10,538.23 ± 40.31 ^e^
	*R. philippinarum*	447.28 ± 9.23 ^h^	138.19 ± 7.75 ^k^	502.89 ± 8.71 ^j^	280.47 ± 4.78 ^b^	2253.84 ± 20.36 ^j^	96.74 ± 1.36 ^h^	5.67 ± 0.01 ^h^	8.78 ± 0.43 ^ef^	3733.85 ± 52.61 ^k^
	*M. galloprovincialis*	4761.02 ± 15.38 ^a^	247.49 ± 6.87 ^j^	317.98 ± 4.83 ^k^	51.83 ± 3.00 ^f^	2250.42 ± 14.46 ^j^	387.59 ± 5.79 ^d^	51.94 ± 0.13 ^a^	15.42 ± 0.58 ^cd^	8083.64 ± 19.71 ^h^
	*C. californiense*	4360.01 ± 30.82 ^b^	377.80 ± 13.29 ^i^	2536.31 ± 19.64 ^c^	82.31 ± 2.26 ^d^	5385.86 ± 28.43 ^c^	627.39 ± 11.05 ^c^	11.60 ± 0.06 ^e^	8.96 ± 0.43 ^ef^	13,390.24 ± 39.22 ^b^
	*S. strictus*	646.03 ± 20.61 ^f^	1504.05 ± 40.35 ^d^	5060.04 ± 15.74 ^a^	32.39 ± 1.05 ^g^	4099.55 ± 16.37 ^f^	630.82 ± 4.63 ^c^	12.84 ± 0.04 ^d^	15.09 ± 0.39 ^cd^	12,000.81 ± 34.96 ^c^
	*C. farreri*	615.08 ± 10.68 ^f^	1088.48 ± 9.80 ^g^	1151.83 ± 4.63 ^g^	65.29 ± 3.69 ^e^	3610.16 ± 15.04 ^g^	-	15.80 ± 0.11 ^c^	10.11 ± 0.26 ^e^	6556.74 ± 13.61 ^i^
	*S. subcrenata*	511.54 ± 5.79 ^g^	2203.31 ± 23.16 ^c^	1962.33 ± 8.52 ^d^	109.83 ± 0.95 ^c^	11,095.26 ± 34.26 ^a^	261.14 ± 7.12 ^e^	8.02 ± 0.03 ^f^	32.94 ± 0.73 ^a^	16,184.39 ± 62.06 ^a^
	*M. veneriformis*	1189.02 ± 15.49 ^d^	3701.68 ± 18.62 ^b^	1680.05 ± 20.33 ^e^	62.24 ± 1.46 ^e^	4708.31 ± 16.69 ^e^	698.87 ± 10.60 ^b^	2.53 ± 0.06 ^j^	27.78 ± 0.84 ^b^	12,070.48 ± 17.52 ^c^
	*M. meretrix*	285.25 ± 11.64 ^j^	4279.10 ± 14.92 ^a^	1425.23 ± 14.86 ^f^	-	2904.50 ± 26.43 ^i^	-	7.06 ± 0.10 ^g^	3.91 ± 0.05 ^g^	8905.05 ± 14.79 ^g^
	*S. constricta*	3224.62 ± 16.81 ^c^	-	3542.85 ± 24.44 ^b^	17.60 ± 0.48 ^h^	3310.77 ± 17.80 ^h^	991.02 ± 17.85 ^a^	17.54 ± 0.39 ^b^	16.62 ± 0.11 ^c^	11,121.02 ± 76.14 ^d^

Different letters within a column indicate significant difference (*p* < 0.05); - not detected.

**Table 10:** EUCs of 13 shellfish meat and their boiling liquids (g MSG/100 g, *n* = 3).

Shellfish Species	EUCs	
	Shellfish Meat	Boiling Liquids
*M. chinensis*	122.8 ± 0.4 ^f^	99.8 ± 0.5 ^h^
*C. sinensis*	93.4 ± 0.6 ^i^	29.1 ± 0.2 ^l^
*C. gigas*	114.9 ± 1.2 ^g^	51.7 ± 0.8 ^j^
*S. broughtonii*	59.4 ± 1.6 ^k^	34.1 ± 0.4 ^k^
*R. philippinarum*	305.2 ± 0.7 ^c^	286.8 ± 0.6 ^b^
*M. galloprovincialis*	450.4 ± 3.2 ^b^	142.1 ± 0.3 ^f^
*C. californiense*	261.9 ± 0.6 ^d^	358.3 ± 0.9 ^a^
*S. strictus*	258.0 ± 5.7 ^d^	140.6 ± 0.2 ^f^
*C. farreri*	102.6 ± 1.1 ^h^	248.4 ± 1.0 ^c^
*S. subcrenata*	84.5 ± 0.5 ^j^	110.5 ± 0.2 ^g^
*M. veneriformis*	165.0 ± 3.0 ^e^	230.9 ± 1.1 ^d^
*M. meretrix*	586.8 ± 1.4 ^a^	65.5 ± 0.7 ^i^
*S. constricta*	62.3 ± 0.1 ^k^	187.5 ± 1.0 ^e^

Different letters within a column indicate significant differences (*p* < 0.05); equivalent umami concentration (EUC).

**Table 11:** Eigenvalue, variance contribution rate, and cumulative variance contribution rate of the four principal components.

Index	PC1	PC2	PC3	PC4
EAA/TAA	0.909	−0.283	−0.161	−0.134
EAA/NEAA	0.904	−0.288	−0.173	−0.141
Taurine	0.687	−0.521	0.105	−0.361
Ash	−0.622	−0.345	0.532	−0.049
Meat yield	−0.221	0.796	−0.026	−0.510
Protein	0.534	0.731	0.157	−0.133
n3/n6	0.334	0.548	0.098	0.535
Fat	0.286	0.304	−0.779	0.348
PUFA	0.451	0.165	0.665	0.434
Polysaccharide	−0.378	−0.495	−0.649	0.293
EUC	0.223	−0.380	0.524	0.277
Eigenvalue	3.425	2.532	2.129	1.207
Variance contribution rate (%)	31.137	23.014	19.357	10.970
cumulative variance contribution rate (%)	31.137	54.151	73.508	84.478

**Table 12:** Comprehensive quality scores of 13 shellfish species.

Shellfish Species	Scores of First Principal Components (F1)	Scores of Second Principal Components (F2)	Scores of Third Principal Components (F3)	Scores of Fourth Principal Components (F4)	Comprehensive Score (F)	Rank
*M. chinensis*	−0.30	1.57	1.89	−1.80	0.44	4
*C. sinensis*	−3.17	−1.43	1.22	−0.89	−1.18	11
*C. gigas*	0.92	−1.76	−1.32	1.02	−0.26	9
*S. broughtonii*	2.56	0.54	−1.57	0.23	0.64	2
*R. philippinarum*	−0.10	−0.35	0.19	−0.05	−0.08	7
*M. galloprovincialis*	1.44	−0.95	1.37	0.37	0.54	3
*C. californiense*	1.27	2.08	1.05	0.92	1.18	1
*S. strictus*	0.35	1.16	0.16	−0.93	0.30	5
*C. farreri*	−0.21	2.64	−0.13	1.15	0.64	2
*S. subcrenata*	2.18	−1.21	−1.31	−1.88	−0.06	6
*M. veneriformis*	−2.23	−0.28	0.55	1.42	−0.50	10
*M. meretrix*	0.41	−2.63	1.08	0.75	−0.18	8
*S. constricta*	−3.13	0.61	−3.18	−0.30	−1.48	12

## Data Availability

The data presented in this study are available on request from the corresponding author.
